# Kinetostatic Modeling and Workspace Analysis of Redundant Actuated *n*-4R Compliant Parallel Pointing Mechanism

**DOI:** 10.3390/mi16040478

**Published:** 2025-04-18

**Authors:** Jun Ren, Yikang Shu, Youwei Lin

**Affiliations:** Hubei Key Laboratory of Modern Manufacturing Quantity Engineering, School of Mechanical Engineering, Hubei University of Technology, Wuhan 430068, China; 102200008@hbut.edu.cn (Y.S.); 102310073@hbut.edu.cn (Y.L.)

**Keywords:** compliant parallel mechanism (CPM), kinetostatic, redundant actuation, flexible hinge, workspace

## Abstract

The workspace of the compliant parallel mechanism (CPM) is generally limited due to the small deformation range of flexible hinges, which are usually at the micro/nano scale. This paper takes the 2-DOFs *n*-4R compliant parallel pointing mechanism (*n*-4R CPPM) as the object and optimizes the workspace performance of the mechanism through redundant actuation, aiming to maximize the workspace. First, the kinetostatic model and the flexible hinge displacement model of the redundant actuated *n*-4R CPPM are established, successively. The former model reveals the relationships between the output displacements and the input forces/displacements, while the latter relates the flexible hinge deformation and the input forces/displacements. Second, a space pointing trajectory is chosen to validate the accuracy of the kinetostatic model of the redundant actuated 3-4R CPPM through finite element (FE) simulation. The results show that the relative error between the analytical and the FE results does not exceed 2.1%, and the high consistency indicates the accuracy of the kinetostatic model. Finally, the workspace performance of the 3-4R and 4-4R CPPMs is successively optimized through redundant actuation. The results indicate that, compared with the non-redundant actuation case, the workspace can be effectively enlarged and become more symmetric by means of the redundant actuation. The maximum achievable pitch angle *ψ_a_* and the *y*-direction motion range of the mobile platform both increase by 100%. Moreover, it is shown that the workspace in the non-redundant actuated case is a subset of the workspace in the redundant actuated case, and the position-workspace shape changes from planar to 3-D.

## 1. Introduction

Compliant parallel mechanisms (CPMs) integrate the characteristics of compliant mechanism, such as high precision, no friction, and no lubrication [1,2,3], as well as the advantages of parallel mechanism, like quick response and large bearing capacity [4,5,6]. Therefore, they are widely used in various fields, including cell micro-injection [7], precise assembly [8], micro electromechanical systems [9], micro/nano-scratching [10], and atomic force microscopy [11]. Since the motion of these mechanisms is achieved through the elastic deformation of flexible hinges, whose deformation range is typically confined to the micro/nano scale, this greatly restricts the motion range of the mechanisms. As a result, CPMs often encounter the disadvantage of small workspace, which to some extent limits their applicability [3,12,13].

Some scholars have attempted to increase the limited motion range of the mechanisms by adding displacement amplifiers at either the input or output ends of the mechanisms. Chen et al. [14] proposed an orthogonal displacement amplifier applicable to a compact micro-gripper system and established the computational model of displacement amplification. The results show that the displacement amplifier could amplify the output displacement of the micro-gripper system by more than 4.52 times and ensure precise orthogonal motion. Ling et al. [15] proposed a hybrid displacement-bending amplifier. By combining a lever-type and a semi-bridge displacement amplifier, the amplification performance was enhanced, enabling the output displacement of the mechanism to be amplified 6 times. Chen et al. [16] proposed a micro-gripper with a high magnification ratio and three-stage displacement amplification, and the mechanical model of displacement amplification was established. Through optimized compensation of displacement amplification, the amplification factor of the micro-gripper reached 51.2. In addition, some scholars have increased the workspace of CPMs through structural optimization. Yun et al. [17], based on the 3-PUPU CPM, aimed to maximize the workspace and optimized the structural parameters, such as the length of the branch, the distribution radius of the flexible hinges, and the length of the flexible hinges. The results showed that the size of workspace was increased by 72.63%. Ren et al. [18] proposed a generalized 3-PSS CPM with variable guide-rail inclination angles. The results showed that the workspace of the mechanism could be increased by choosing a large guide-rail inclination angle. Although either adding displacement amplifiers or structural optimization can effectively increase the motion range of the mechanism, the cost is a decrease in the motion resolution and dynamic performance of the mechanism [19]. These additions may also lead to structural changes in the mechanism, causing the mechanism to fail to meet the design requirements and affecting its applicability and reliability in practical applications.

Some scholars also attempted to expand the workspace of the mechanisms through redundant actuation. Liu et al. [20] proposed a novel 6RPS symmetric parallel mechanism with redundant actuation, and the dimension optimization is operated by considering the transmission performance indexes. The results showed that, compared with the non-redundant actuation case, the better high-performance workspace and accessible workspace could be obtained by means of redundant actuation. Han et al. [21] proposed a redundant actuated 6R parallel mechanism. The results showed that there was no singularity configuration in the 6R parallel mechanism under the redundant actuation case, and the good transmission workspace increased by 136 times. Zhang et al. [22] proposed a redundant actuated 3-DOFs parallel mechanism, and the workspace of the mechanism was solved by a numerical method. The results showed that the size of workspace was approximately 17% higher than that in the non-redundant actuation case. Li et al. [23] proposed a novel 6-DOFs hybrid mechanism, and the lower part of the mechanism consisted of a 3-DOFs redundant actuated mechanism. The results showed that, compared with the non-redundant actuation, the redundant actuated mechanism exhibited a cubic, continuous, uniform, and larger workspace. However, these studies on expanding the workspace of mechanisms through redundant actuation are mainly focused on rigid mechanisms, with little attention paid to compliant mechanisms.

In 2022, Ren et al. [24] proposed a class of flexure-based *n*-4R compliant parallel pointing mechanisms (*n*-4R CPPM) with 2-DOFs. One characteristic of this class of mechanism is that regardless of the number of branches *n* selected (as long as *n* ≥ 3), the degrees of freedom and motion form of the mechanism remain unchanged. Inspired by the employment of redundant actuation in rigid mechanisms to enlarge the workspace, this paper focuses on the typical 3-4R and 4-4R CPPMs in this class of mechanism (*n*-4R CPPM), aiming to expand the workspace through redundant actuation. The advantage of this approach is that it optimizes the workspace from the perspective of the actuation forces control strategy without the structural changes of the original mechanism.

At present, the calculation of the workspace of compliant parallel mechanisms is mainly based on the pseudo-rigid-body model [25,26,27]. This method considers flexible hinges as traditional stiffness hinges, and only the motion in the main functional direction of the hinge is considered during the calculation process, while small deformations in other non-functional directions are ignored. Therefore, to better reveal the displacement of flexible hinges during the mechanism’s motion, the workspace will be calculated based on the kinetostatic model in this paper. This method takes into account the displacement of flexible hinges in all six directions, enabling a more accurate determination of the mechanism’s workspace.

The remaining sections of this paper are organized as follows. Section 2 takes the 3-4R CPPM as an example to introduce the structure of *n*-4R CPPM. In Section 3, the kinetostatic models which relate the input forces/displacements and the output displacements of the redundant actuated *n*-4R CPPM are established. In Section 4, the constraints of the mechanism’s workspace are determined, and the flexible hinge displacement model which relates the input forces/displacements and the flexible hinge displacements of the redundant actuated *n*-4R CPPM is established. In Section 5, the kinetostatic model is validated by FE-analysis using a given oscillation trajectory of the mobile platform of redundant actuated 3-4R CPPM. Then, the effectiveness of the redundant actuation expanding the workspace of the mechanism is verified. Finally, the conclusions are summarized in Section 6.

## 2. Structure Description of the *n*-4R CPPM

**Figure 1:**
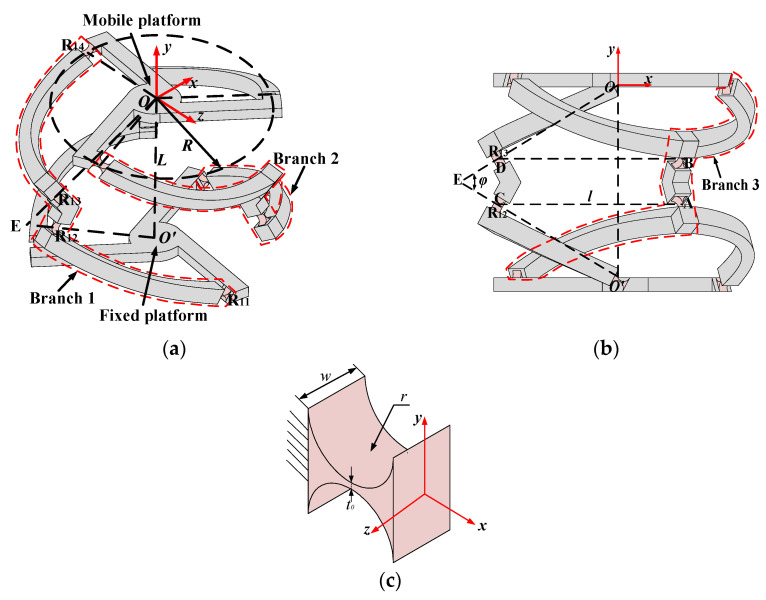
The structure and dimensions of the 4-4R CPPM: (**a**) Structure 1; (**b**) Structure 2; (**c**) Structure parameters of the right-circular flexure hinge.

The *n*-4R CPPM is a 2-DOFs parallel micro-motion turntable that can realize spherical-like motion. The components of this class of mechanism include a fixed platform, a mobile platform, and *n* branches. The fixed platform at the bottom has the same structure as the mobile platform at the top. The *n* branches connecting the fixed and mobile platforms are identical and evenly distributed at 2π/*n* intervals in the circumferential direction. As shown in Figure 1a,b, taking the 3-4R CPPM as an example, each branch is composed of three connecting rods connected in series by four right-circular flexure hinges (hereinafter referred to as flexure hinges). The parameters defining the right-circular flexure hinge are shown in Figure 1c, where the width, radius, and minimum thickness of the right-circular flexure hinge are defined as *w*, *r*, and *t*_0_, respectively. The four flexure hinges in the *i*-th branch are successively defined as R*_i_*_1_, R*_i_*_2_, R*_i_*_3_, and R*_i_*_4_ (*i* = 1…3) from the bottom to top. The intersection of the axes of the four flexible hinges R*_i_*_1_, which are attached to the fixed platform below, is known as the center *O*’ of the fixed platform. The center *O* of the mobile platform is defined as the intersection point of the axes of the four flexible hinges R*_i_*_4_, which is connected to the mobile platform above. The center distance *O*′*O* between the fixed and mobile platforms is set to *L*. The geometric centers of flexible hinges R*_i_*_1_ and R*_i_*_4_ are on the distribution circles of fixed and mobile platforms, respectively. And the distribution radii of fixed and mobile platforms are defined as R. The axes of flexure hinges R_12_ and R_13_ in branch 1 respectively pass through the points *O*′ and *O*, and their intersection point is defined as E. Based on the geometric properties, it is evident that *O*′*E* is equal to *OE*. The angle ∠*OEO*′ between *O*′*E* and *OE* is defined as *φ*. The geometric centers of flexure hinges R_12_ and R_13_ in branch 1 are set to points A and B, respectively. The geometric centers of flexure hinges R_22_ and R_23_ in branch 2 are set to points C and D, respectively. According to the symmetry of the structure, AC is parallel and equivalent to BD. Define AC = BD = *l*.

**Figure 2:**
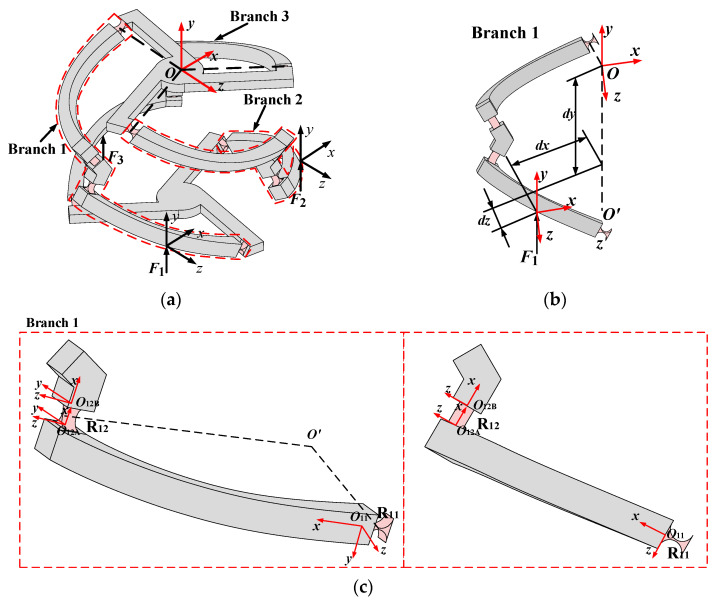
Coordinate frames of the 3-4R CPPM: (**a**) Force loading position; (**b**) Force coordinate frame setting; (**c**) Hinge coordinate frame setting.

As shown in Figure 2a, the 3-4R CPPM is conventionally actuated by two actuators, and the actuation forces ***F***_1_ and ***F***_2_ are respectively exerted on branch 1 and branch 2. The mobile platform has two degrees of freedom in directions of *θ_x_* and *θ_z_*. In this paper, a novel redundant actuated 3-4R CPPM is formed by adding an additional actuation force ***F***_3_ on branch 3. The global coordinate frame *Oxyz* is located at the initial position of the mobile platform center, and the axis of flexible hinge R_14_ is consistent with the *z*-axis of the coordinate frame *Oxyz*. As shown in Figure 2b, Force ***F****_i_* (*i* = 1…3) exerting on the *i*-th branch is subjected to the centroid of the rod connecting flexure hinges R*_i_*_1_ and R*_i_*_2_, at the force coordinate frame *F_i_xyz*, aligned with the *y*-axis of *F_i_xyz*, to distances *dx*, *dy*, and *dz* from the global coordinate frame *Oxyz*. The orientations of all the above force coordinate frames are consistent with that of the global coordinate frame. As can be seen from the above, for the flexible hinge R*_i_*_1_ (*i* = 1…3), one end is fixed on the fixed platform, which is referred to as the fixed end, while the other end is connected to the connecting rod, known as the free end. For the flexible hinge R*_i_*_2_, both ends are defined as free ends because both of them are connected to the rods. As shown in Figure 2c, taking branch 1 as an example, the hinge coordinate frame *O*_11_*xyz* is located at the center of the free end of the flexible hinge R_11_, and the hinge coordinate frames *O*_12A_*xyz* and *O*_12B_*xyz* are located at the centers of the free ends of the flexible hinge R_12_ near the fixed platform and the mobile platform, respectively. The *z*-axis of each hinge coordinate frame is parallel to the axis of the flexible hinge. In addition, the hinge coordinate frames at the free end of the flexible hinges R*_i_*_1_ and R*_i_*_2_ are located in the same way as that of the hinge coordinate frame of the flexible hinges R_11_ and R_12_, respectively.

## 3. Kinetostatic Modeling of the Redundant Actuated *n*-4R CPPM

For the convenience of calculating the workspace later, the kinetostatic model of the redundant actuated *n*-4R CPPM is first modeled in this section. We take the redundant actuated 3-4R CPPM as the object to model its kinetostatic in Section 3.1, and then extend it to the case of the redundant actuated *n*-4R CPPM in Section 3.2.

### 3.1. Kinetostatic Model of Redundant Actuated 3-4R CPPM

#### 3.1.1. The Relationship Between Input Force and Output Displacement

The generalized forces (***F***_1_, ***F***_2_, and ***F***_3_) and output displacement ***U***_3-4R_ are defined as:

(1) $$\left\{\begin{array}{c} F_{i}={\left[\begin{array}{cccccc} m_{i,x} & m_{i,y} & m_{i,z} & f_{i,x} & f_{i,y} & f_{i,z} \end{array}\right]}^{T}\\ U_{3\text{-}4R}={\left[\begin{array}{cccccc} \theta_{x} & \theta_{y} & \theta_{z} & \delta_{x} & \delta_{y} & \delta_{z} \end{array}\right]}^{T} \end{array}\right.$$

where *i* = 1, 2, 3 is the order number of branches.

**Figure 3:**
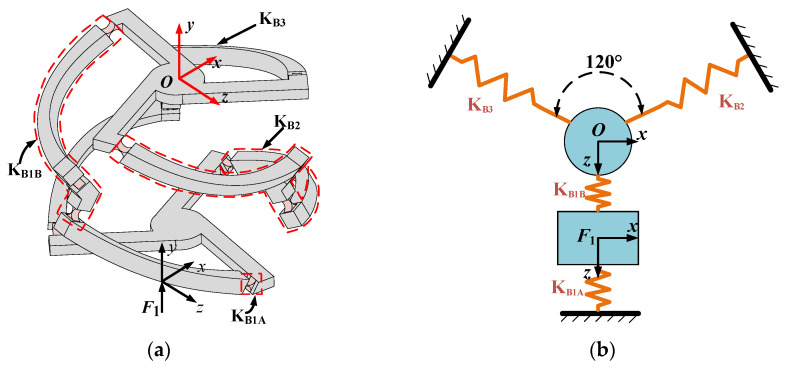
3-4R CPPM subjected to force ***F***_1_: (**a**) Simplification of equivalent stiffness; (**b**) Equivalent Spring system.

Assuming that the deformation of the mechanism is within the linear range, the output displacement ***U***_3-4R_ can be regarded as the superposition of the displacements generated by each individual input force acting separately. Therefore, it is necessary to first analyze the displacement generated by the mobile platform under the action of a single input force. For instance, consider that the 3-4R CPPM is only subject to the input force ***F***_1_, as shown in Figure 3a. For the convenience of analysis, the concept of equivalent stiffness [28,29] is introduced, and the redundant actuated 3-4R CPPM is simplified as an equivalent spring system, as shown in Figure 3b. Based on Hooke’s law, the governing equation of the elastic deformation of the system can be constructed as:

(2) $$\left[\begin{array}{cc} {\left(K_{OO}\right)}_{F_{1}} & K_{OF_{1}}\\ K_{F_{1}O} & K_{F_{1}F_{1}} \end{array}\right]\left[\begin{array}{c} U_{1}\\ U_{F_{1}} \end{array}\right]=\left[\begin{array}{c} F_{U}\\ F_{1} \end{array}\right]$$

where ***U***_1_ denotes the output displacement of the mobile platform with respect to global coordinate frame *Oxyz*, $U_{F_{1}}$ denotes the displacement of with respect to the force coordinate frame *F*_1_*xyz*, and ***F****_U_* denotes the force exerted on the mobile platform center.

The stiffness matrices in Equation (2) can be calculated as:

(3) $$\left\{\begin{array}{c} {\left(K_{OO}\right)}_{F_{1}}=K_{B1B}^{O}+K_{B2}^{O}+K_{B3}^{O},K_{F_{1}F_{1}}=K_{B1A}^{F_{1}}+K_{B1B}^{F_{1}}\\ {Κ}_{OF_{1}}=-{\left(Ad_{O}^{O}\right)}^{-T}\left[K_{B1B}^{O}\right]{\left(Ad_{O}^{F_{1}}\right)}^{-1},K_{F_{1}O}=-{\left(Ad_{O}^{F_{1}}\right)}^{-T}\left[K_{B1B}^{O}\right]{\left(Ad_{O}^{O}\right)}^{-1} \end{array}\right.$$

where ***K****_B_*_1*A*_ and ***K****_B_*_1*B*_ denote the equivalent stiffness matrices of the segments between the acting position on branch 1 and the fixed and mobile platforms, respectively. ***K****_B_*_2_ and ***K****_B_*_3_ respectively denote the equivalent stiffness matrices of branch 2 and 3, as shown in Figure 3. The superscript *O* and *F*_1_ indicate the location of the stiffness matrices with respect to coordinate frame *Oxyz* and *F*_1_*xyz*, respectively, and the dimensions are 6 × 6. $Ad_{O}^{O}$ and $Ad_{O}^{F_{1}}$ are the adjoint matrices of coordinate transformation. $Ad_{O}^{F_{1}}$ represents the coordinate transformation from global coordinate frame *Oxyz* to force coordinate frame *F*_1_*xyz*, which can be computed by:

$$Ad_{O}^{O}=I_{6\times 6},\;Ad_{O}^{F_{1}}=\left[\begin{array}{cc} R_{O}^{F_{1}} & 0\\ T_{O}^{F_{1}}\cdot R_{O}^{F_{1}} & R_{O}^{F_{1}} \end{array}\right],\;\mathrm{where}\;T_{O}^{F_{1}}=\left[\begin{array}{ccc} 0 & d_{3} & d_{2}\\ -d_{3} & 0 & -d_{1}\\ -d_{2} & d_{1} & 0 \end{array}\right],\;R_{O}^{F_{1}}=I_{3\times 3}$$

The matrices in Equation (3) are computed by:

(4) $$\left\{\begin{array}{c} K_{B1A}^{O}={\left(C_{11}\right)}^{-1},K_{B1B}^{O}={\left(C_{12}+C_{13}+C_{14}\right)}^{-1},{\begin{array}{c} K_{B2}^{O}=\left(C_{2}\right) \end{array}}^{-1},{\begin{array}{c} K_{B3}^{O}=\left(C_{3}\right) \end{array}}^{-1}\\ K_{B1A}^{F_{1}}={\left(Ad_{O}^{F_{1}}{\left(K_{B1A}^{O}\right)}^{-1}{\left(Ad_{O}^{F_{1}}\right)}^{T}\right)}^{-1},K_{B1B}^{F_{1}}={\left(Ad_{O}^{F_{1}}{\left(K_{B1B}^{O}\right)}^{-1}{\left(Ad_{O}^{F_{1}}\right)}^{T}\right)}^{-1} \end{array}\right.$$

where ***C***_1*i*_ (*i* = 1, 2, 3, 4) and ***C****_i_* (*i* = 1, 2, 3) represent the compliant matrices of the flexible hinge R_1*i*_ and the branch *i* in the global coordinate frame *Oxyz*, respectively. It should be noted that the compliant matrices ***C***_1*i*_ and ***C****_i_* can be calculated using Formula (2) given in the literature [24].

The displacement $U_{1}$ and $U_{F_{1}}$ can be computed from Equation (2) as:

(5) $$U_{1}=-{\left({\left(K_{OO}\right)}_{F_{1}}-K_{OF_{1}}K_{F_{1}F_{1}}^{-1}K_{F_{1}O}\right)}^{-1}\cdot \left(\left(K_{OF_{1}}K_{F_{1}F_{1}}^{-1}\right)\cdot F_{1}-F_{U}\right)$$

(6) $$U_{F_{1}}={\left(K_{F_{1}F_{1}}-K_{F_{1}O}{\left(K_{OO}\right)}_{F_{1}}^{-1}K_{OF_{1}}\right)}^{-1}\cdot \left(F_{1}-\left(K_{F_{1}O}{\left(K_{OO}\right)}_{F_{1}}^{-1}\right)\cdot F_{U}\right)$$

Assuming no external force exerted on the mobile platform center, ***F****_U_* = 0. Thus, Equations (5) and (6) can be further simplified to:

(7) $$U_{1}=-{\left({\left(K_{OO}\right)}_{F_{1}}-K_{OF_{1}}K_{F_{1}F_{1}}^{-1}K_{F_{1}O}\right)}^{-1}\left(K_{OF_{1}}K_{F_{1}F_{1}}^{-1}\right)F_{1}$$

(8) $$U_{F_{1}}={\left(K_{F_{1}F_{1}}-K_{F_{1}O}{\left(K_{OO}\right)}_{F_{1}}^{-1}K_{OF_{1}}\right)}^{-1}F_{1}$$

Assuming no external force exerted on the branch 1, ***F***_1_ = 0. Equation (6) can be further simplified to:

(9) $$U_{F_{1}}=C_{O}^{F_{1}}\cdot F_{U},C_{O}^{F_{1}}=-{\left(K_{F_{1}F_{1}}-K_{F_{1}O}{\left(K_{OO}\right)}_{F_{1}}^{-1}K_{OF_{1}}\right)}^{-1}K_{F_{1}O}{\left(K_{OO}\right)}_{F_{1}}^{-1}$$

Equation (7) expresses the displacement of the mobile platform ***U***_1_ caused by the force ***F***_1_. Similarly, to describe the displacement of ***U****_i_* (*i* = 1, 2, 3) resulting from any force ***F****_i_* exerted on the *i*-th branch in the terms of compliance matrix, Equation (10) yields:

(10) $$U_{i}=C_{F_{i}}^{O}F_{i},C_{F_{i}}^{O}=-{\left({\left(K_{OO}\right)}_{F_{i}}-K_{OF_{i}}K_{F_{i}F_{i}}^{-1}K_{F_{i}O}\right)}^{-1}\left(K_{OF_{i}}K_{F_{i}F_{i}}^{-1}\right)$$

When all the forces, ***F***_1_, ***F***_2_, and ***F***_3_, act on the mobile platform at the same time, the total displacement ***U***_3-4R_ can be considered as the superposition of the displacements ***U***_1_, ***U***_2_ and ***U***_3_. Hence, the total displacement of the mobile platform can be computed by:

(11) $$U_{3\text{-}4R}=C_{Total}^{O}F_{Total},\;\mathrm{where}\;C_{Total}^{O}=\left[\begin{array}{ccc} C_{F_{1}}^{O} & C_{F_{2}}^{O} & C_{F_{3}}^{O} \end{array}\right]\;\mathrm{and}\;F_{Total}=\left[\begin{array}{c} F_{1}\\ F_{2}\\ F_{3} \end{array}\right]$$

So far, the kinetostatic model relating the generalized input forces and the generalized output displacements of the redundant actuated 3-4R CPPM is established by Equation (11). For the convenience of practical application, a simplified kinetostatic model directly relating the output angular displacements (*θ_x_* and *θ_z_*) in the direction of two degrees of freedom with the input component forces *f*_*i*,*y*_ (*i* = 1, 2, 3) along the y-direction is further extracted from the Equation (11), as shown in Equation (12).

(12) $$\left[\begin{array}{c} \theta_{x}\\ \theta_{z} \end{array}\right]=\left[\begin{array}{ccc} {\left(C_{\theta_{x}}\right)}_{f_{1,y}} & {\left(C_{\theta_{x}}\right)}_{f_{2,y}} & {\left(C_{\theta_{x}}\right)}_{f_{3,y}}\\ {\left(C_{\theta_{z}}\right)}_{f_{1,y}} & {\left(C_{\theta_{z}}\right)}_{f_{2,y}} & {\left(C_{\theta_{z}}\right)}_{f_{3,y}} \end{array}\right]\left[\begin{array}{c} f_{1,y}\\ f_{2,y}\\ f_{3,y} \end{array}\right]$$

where

(13) $$\left[\begin{array}{ccc} {\left(C_{\theta_{x}}\right)}_{f_{1,y}} & {\left(C_{\theta_{x}}\right)}_{f_{2,y}} & {\left(C_{\theta_{x}}\right)}_{f_{3,y}}\\ {\left(C_{\theta_{z}}\right)}_{f_{1,y}} & {\left(C_{\theta_{z}}\right)}_{f_{2,y}} & {\left(C_{\theta_{z}}\right)}_{f_{3,y}} \end{array}\right]=\left[\begin{array}{ccc} {\left(C_{F_{1}}^{O}\right)}_{\left[r1,3,\;c5\right]} & {\left(C_{F_{2}}^{O}\right)}_{\left[r1,3,\;c5\right]} & {\left(C_{F_{3}}^{O}\right)}_{\left[r1,3,\;c5\right]} \end{array}\right]$$

where *θ_x_* and *θ_z_* represent the output angular displacements of the mobile platform rotating around the *x*-axis and *z*-axis of the global coordinate frame *Oxyz*, respectively. Matrix ${\left(C_{F_{1}}^{O}\right)}_{\left[r1,3,\;c5\right]}$ is constructed with the elements of the first and third rows of the fifth column of matrix $C_{F_{1}}^{O}$. The matrix element ${\left(C_{\theta_{x}}\right)}_{f_{1,y}}$ relates the output angular displacement in *θ_x_*-direction of the mobile platform with the input force acting in *y*-direction of the force coordinate frame *F*_1_*xyz*.

#### 3.1.2. The Relationship Between Input Displacement and Output Displacement

Equation (11) reveals the relationship between all input forces and the resulting output displacements of the mobile platform. In practical application, however, sometimes the actuators take the displacement rather than the force as the input. Therefore, it is essential to further derive the relationship relating the input displacements and the output displacements. The corresponding input displacement is the equivalent input displacement based on the input force.

Equation (8) describes the input displacement $U_{F_{1}}$ at the force coordinate frame *F*_1_*xyz* produced by the input force ***F***_1_ acting alone. Since total input displacement $U_{F_{1}\_total}$ at the force coordinate frame *F*_1_*xyz* results from the combined action of all input forces ***F***_1_, ***F***_2_ and ***F***_3_, it needs to derive the expressions that relate the input displacement $U_{F_{1}}$ with the other input forces ***F****_i_* (*i* = 2, 3). Similar to Equation (2), the compensation equation of input force ***F****_i_* to input displacement $U_{F_{1}}$ is constructed according to Hooke’s law as follows:

(14) $$\left[\begin{array}{c} F_{1}\\ F_{U}\\ F_{i} \end{array}\right]=\left[\begin{array}{ccc} K_{F_{1}F_{1}} & K_{F_{1}O} & 0\\ {Κ}_{OF_{1}} & {\left(K_{OO}\right)}_{F_{i}} & {Κ}_{OF_{i}}\\ 0 & K_{F_{i}O} & K_{F_{i}F_{i}} \end{array}\right]\left[\begin{array}{c} U_{F_{1}}\\ U_{1}\\ U_{F_{i}} \end{array}\right]$$

where ***F****_i_* represents the force exerted on the branch *i*, and $U_{F_{i}}$ represents the displacement at the force coordinate frame *F_i_xyz*, and the remaining elements are consistent with the definition in Equation (2). The stiffness matrices ${\left(K_{OO}\right)}_{F_{i}}$, ${Κ}_{OF_{i}}$, $K_{F_{i}O}$ and $K_{F_{i}F_{i}}$ can be calculated as:

(15) $$\left\{\begin{array}{c} {\left(K_{OO}\right)}_{F_{i}}=K_{B1B}^{O}+K_{BiB}^{O}+K_{3\text{-}4R}-K_{B1}^{O}-K_{Bi}^{O},K_{F_{i}F_{i}}=K_{BiA}^{F_{i}}+K_{BiB}^{F_{i}}\\ {Κ}_{OF_{i}}=-{\left(Ad_{O}^{O}\right)}^{-T}\left[K_{BiB}^{O}\right]{\left(Ad_{O}^{F_{i}}\right)}^{-1},K_{F_{i}O}=-{\left(Ad_{O}^{F_{i}}\right)}^{-T}\left[K_{BiB}^{O}\right]{\left(Ad_{O}^{O}\right)}^{-1} \end{array}\right.$$

where $Ad_{O}^{F_{i}}$ represents the coordinate transformation from the global coordinate frame *Oxyz* to the force coordinate frame *F_i_xyz*, which is calculated as:

(16) $$Ad_{O}^{F_{i}}=Ad_{2(i-1)\pi /n}\left[Ad_{O}^{F_{1}}\right]{\left(Ad_{2(i-1)\pi /n}\right)}^{T},Ad_{2(i-1)\pi /n}=\left[\begin{array}{cc} R_{y,2(i-1)\pi /n} & 0\\ 0 & R_{y,2(i-1)\pi /n} \end{array}\right]$$

where $Ad_{2(i-1)\pi /n}$ is the adjoint transformation matrix. $R_{y,2(i-1)\pi /n}$ represents the rotation matrix about the *y*-axis of the global coordinate frame *Oxyz*, and *n* = 3.

The stiffness matrices in Equation (15) are defined by:

(17) $$\left\{\begin{array}{c} K_{BiA}^{O}={\left(Ad_{2(i-1)\pi /3}\right)}^{-T}K_{B1A}^{O}{\left(Ad_{2(i-1)\pi /3}\right)}^{-1},K_{BiA}^{F_{i}}={\left(Ad_{O}^{F_{i}}\right)}^{-T}K_{BiA}^{O}{\left(Ad_{O}^{F_{i}}\right)}^{-1}\\ K_{BiB}^{O}={\left(Ad_{2(i-1)\pi /3}\right)}^{-T}K_{B1B}^{O}{\left(Ad_{2(i-1)\pi /3}\right)}^{-1},K_{BiB}^{F_{i}}={\left(Ad_{O}^{F_{i}}\right)}^{-T}K_{BiB}^{O}{\left(Ad_{O}^{F_{i}}\right)}^{-1}\\ K_{3\text{-}4R}={\left(C_{3\text{-}4R}\right)}^{-1},{\begin{array}{c} K_{B1}^{O}=\left(C_{1}\right) \end{array}}^{-1} \end{array}\right.$$

where ***C***_3-4R_ represents the compliant matrix of 3-4R CPPM and can be calculated using Formula (6) in ref [24].

From Equation (14), one can obtain:

(18) $$U_{F_{1}}=C_{F_{i}}^{F_{1}}F_{i}\begin{array}{cc} & \left(i=2,3\right) \end{array}$$

where $C_{F_{i}}^{F_{1}}={\left(K_{F_{1}F_{1}}-K_{F_{1}O}{\left(K_{R_{i}}\right)}^{-1}{Κ}_{OF_{1}}\right)}^{-1}\left(K_{F_{1}O}{\left(K_{R_{i}}\right)}^{-1}{Κ}_{OF_{i}}{\left(K_{F_{i}F_{i}}\right)}^{-1}\right)$, in which $K_{R_{i}}={\left(K_{OO}\right)}_{F_{i}}-{Κ}_{OF_{i}}{\left(K_{F_{i}F_{i}}\right)}^{-1}K_{F_{i}O}$. When *i* = 1, $C_{F_{1}}^{F_{1}}$ can be obtained from Equation (8):

(19) $$C_{F_{1}}^{F_{1}}={\left(K_{F_{1}F_{1}}-K_{F_{1}O}{\left(K_{OO}\right)}_{F_{1}}^{-1}K_{OF_{1}}\right)}^{-1}$$

Since the total input displacement $U_{F_{1}\_total}$ at the force coordinate frame *F*_1_*xyz* can be regarded as the superposition of the input displacements caused by the input forces ***F***_1_, ***F***_2_ and ***F***_3_ acting alone, one can obtain:

(20) $$U_{F_{1}\_total}=\left[\begin{array}{ccc} C_{F_{1}}^{F_{1}} & C_{F_{2}}^{F_{1}} & C_{F_{3}}^{F_{1}} \end{array}\right]\left[\begin{array}{c} F_{1}\\ F_{2}\\ F_{3} \end{array}\right]$$

Similar to Equation (20), the total input displacement $U_{F_{i}\_total}$ at the force coordinate frame *F_i_xyz* due to the input forces ***F***_1_, ***F***_2_ and ***F***_3_ can be obtained as:

(21) $$U_{F_{i}\_total}=\left[\begin{array}{ccc} C_{F_{1}}^{F_{i}} & C_{F_{2}}^{F_{i}} & C_{F_{3}}^{F_{i}} \end{array}\right]\left[\begin{array}{c} F_{1}\\ F_{2}\\ F_{3} \end{array}\right]\begin{array}{cc} & \left(i=1,2,3\right) \end{array}$$

Due to the symmetry of the structure, compliance matrices $C_{F_{1}}^{F_{i}}$, $C_{F_{2}}^{F_{i}}$ and $C_{F_{3}}^{F_{i}}$ (*i* = 2, 3) can be directly obtained using the results of the compliance matrices $C_{F_{1}}^{F_{1}}$, $C_{F_{2}}^{F_{1}}$ and $C_{F_{3}}^{F_{1}}$, as shown in Equation (22).

(22) $$\left\{\begin{array}{c} C_{F_{1}}^{F_{2}}=C_{F_{3}}^{F_{1}},C_{F_{2}}^{F_{2}}=C_{F_{1}}^{F_{1}},C_{F_{3}}^{F_{2}}=C_{F_{2}}^{F_{1}}\\ C_{F_{1}}^{F_{3}}=C_{F_{2}}^{F_{1}},C_{F_{2}}^{F_{3}}=C_{F_{3}}^{F_{1}},C_{F_{3}}^{F_{3}}=C_{F_{1}}^{F_{1}} \end{array}\right.$$

Then, the input displacement $U_{F_{i}\_total}$ at the force coordinate frame *F_i_xyz* generated by the action of the three input forces ***F***_1_, ***F***_2_, ***F***_3_ can be obtained by rearranging Equations (21) and (22), as shown in Equation (23).

(23) $$\left[\begin{array}{c} U_{F_{1}\_total}\\ U_{F_{2}\_total}\\ U_{F_{3}\_total} \end{array}\right]=\left[\begin{array}{ccc} C_{F_{1}}^{F_{1}} & C_{F_{2}}^{F_{1}} & C_{F_{3}}^{F_{1}}\\ C_{F_{1}}^{F_{2}} & C_{F_{2}}^{F_{2}} & C_{F_{3}}^{F_{2}}\\ C_{F_{1}}^{F_{3}} & C_{F_{2}}^{F_{3}} & C_{F_{3}}^{F_{3}} \end{array}\right]\left[\begin{array}{c} F_{1}\\ F_{2}\\ F_{3} \end{array}\right]$$

By substituting Equation (23) into (11), the relationship between the input displacements $U_{F_{i}\_total}$ (*i* = 1, 2, 3) at each force coordinate frame *F_i_xyz* and the output displacement ***U***_3-4R_ of the mobile platform can be calculated as:

(24) $$U_{3\text{-}4R}=\left[\begin{array}{ccc} C_{F_{1}}^{O} & C_{F_{2}}^{O} & C_{F_{3}}^{O} \end{array}\right]{\left[\begin{array}{ccc} C_{F_{1}}^{F_{1}} & C_{F_{2}}^{F_{1}} & C_{F_{3}}^{F_{1}}\\ C_{F_{1}}^{F_{2}} & C_{F_{2}}^{F_{2}} & C_{F_{3}}^{F_{2}}\\ C_{F_{1}}^{F_{3}} & C_{F_{2}}^{F_{3}} & C_{F_{3}}^{F_{3}} \end{array}\right]}^{-1}\left[\begin{array}{c} U_{F_{1}\_total}\\ U_{F_{2}\_total}\\ U_{F_{3}\_total} \end{array}\right]$$

Furtherly, a simplified model directly relating the input component force *f*_*i*,*y*_ (*i* = 1, 2, 3) acting in the *y*-direction and the resulting input displacement *d_i_* (*i* = 1, 2, 3) along with the *y*-direction can be extracted from Equation (23), as shown in Equation (25).

(25) $$\left[\begin{array}{c} d_{1}\\ d_{2}\\ d_{3} \end{array}\right]=\left[\begin{array}{ccc} {\left(C_{F_{1}}^{F_{1}}\right)}_{\left[r5,\;c5\right]} & {\left(C_{F_{2}}^{F_{1}}\right)}_{\left[r5,\;c5\right]} & {\left(C_{F_{3}}^{F_{1}}\right)}_{\left[r5,\;c5\right]}\\ {\left(C_{F_{1}}^{F_{2}}\right)}_{\left[r5,\;c5\right]} & {\left(C_{F_{2}}^{F_{2}}\right)}_{\left[r5,\;c5\right]} & {\left(C_{F_{3}}^{F_{2}}\right)}_{\left[r5,\;c5\right]}\\ {\left(C_{F_{1}}^{F_{3}}\right)}_{\left[r5,\;c5\right]} & {\left(C_{F_{2}}^{F_{3}}\right)}_{\left[r5,\;c5\right]} & {\left(C_{F_{3}}^{F_{3}}\right)}_{\left[r5,\;c5\right]} \end{array}\right]\left[\begin{array}{c} f_{1,y}\\ f_{2,y}\\ f_{3,y} \end{array}\right]$$

where ${\left(C_{F_{1}}^{F_{1}}\right)}_{\left[r5,c5\right]}$ represents the element of the fifth column of the fifth row of the compliance matrix $C_{F_{1}}^{F_{1}}$, and the definitions of the remaining elements in the equation are similar.

By substituting Equation (25) into (12), the output angular displacements (*θ_x_* and *θ_z_*) of the mobile platform can be directly described by the three input displacements *d_i_* of the actuators, as shown in Equation (26).

(26) $$\left[\begin{array}{c} \theta_{x}\\ \theta_{z} \end{array}\right]=C_{f}\cdot C_{d}\cdot \left[\begin{array}{c} d_{1}\\ d_{2}\\ d_{3} \end{array}\right]$$

where:

(27) $$\begin{array}{l} C_{f}=\left[\begin{array}{ccc} {\left(C_{\theta_{x}}\right)}_{f_{1,y}} & {\left(C_{\theta_{x}}\right)}_{f_{2,y}} & {\left(C_{\theta_{x}}\right)}_{f_{3,y}}\\ {\left(C_{\theta_{z}}\right)}_{f_{1,y}} & {\left(C_{\theta_{z}}\right)}_{f_{2,y}} & {\left(C_{\theta_{z}}\right)}_{f_{3,y}} \end{array}\right]\\ C_{d}={\left[\begin{array}{ccc} {\left(C_{F_{1}}^{F_{1}}\right)}_{\left[r5,\;c5\right]} & {\left(C_{F_{2}}^{F_{1}}\right)}_{\left[r5,\;c5\right]} & {\left(C_{F_{3}}^{F_{1}}\right)}_{\left[r5,\;c5\right]}\\ {\left(C_{F_{1}}^{F_{2}}\right)}_{\left[r5,\;c5\right]} & {\left(C_{F_{2}}^{F_{2}}\right)}_{\left[r5,\;c5\right]} & {\left(C_{F_{3}}^{F_{2}}\right)}_{\left[r5,\;c5\right]}\\ {\left(C_{F_{1}}^{F_{3}}\right)}_{\left[r5,\;c5\right]} & {\left(C_{F_{2}}^{F_{3}}\right)}_{\left[r5,\;c5\right]} & {\left(C_{F_{3}}^{F_{3}}\right)}_{\left[r5,\;c5\right]} \end{array}\right]}^{-1} \end{array}$$

### 3.2. Kinetostatic Model of Redundant Actuated n-4R CPPM

In this section, the kinetostatic model is further extended from redundant actuated 3-4R to a class of redundant actuated *n*-4R CPPM. The number of actuation forces of the redundant actuated *n*-4R CPPM is defined as *j* (*j* = 3…*n*). It can be seen from Equation (3) that only the parameters ***K****_B_*_2_ and ***K****_B_*_3_ are associated with the number of mechanism’s branches. Therefore, in order to avoid repetition, this section directly gives relevant conclusions. The mapping matrix between the force ***F***_1_ applied to the branch 1 and the displacement ***U***_1_ of the mobile platform is as follows:

(28) $$C_{F_{1}}^{O}=-{\left({\left(K_{OO}\right)}_{F_{1}}-K_{OF_{1}}K_{F_{1}F_{1}}^{-1}K_{F_{1}O}\right)}^{-1}\left(K_{OF_{1}}K_{F_{1}F_{1}}^{-1}\right)$$

where:

(29) $$\left\{\begin{array}{c} {\left(K_{OO}\right)}_{F_{1}}=K_{B1B}^{O}+K_{n\text{-}4R}-K_{B1}^{O},K_{F_{1}F_{1}}=K_{B1A}^{F_{1}}+K_{B1B}^{F_{1}}\\ {Κ}_{OF_{1}}=-{\left(Ad_{O}^{O}\right)}^{-T}\left[K_{B1B}^{O}\right]{\left(Ad_{O}^{F_{1}}\right)}^{-1},K_{F_{1}O}=-{\left(Ad_{O}^{F_{1}}\right)}^{-T}\left[K_{B1B}^{O}\right]{\left(Ad_{O}^{O}\right)}^{-1} \end{array}\right.$$

where ***K***_n-4R_ represents the stiffness matrix of *n*-4R CPPM. The remaining stiffness matrices can be obtained from Equation (3).

Since the *n* branches of the redundant actuated *n*-4R CPPM are evenly distributed at the same location in the circumferential direction, the mapping matrix between the input force ***F****_i_* (*i* = 2, …, *j*) and the output displacement can be obtained by rotating the matrix $C_{F_{1}}^{O}$ by 2 (*i* − 1)π/*n* around the *y*-axis of the global coordinate frame *Oxyz*.

(30) $$C_{F_{i}}^{O}={\left(Ad_{2(i-1)\pi /n}\right)}^{-T}C_{F_{1}}^{O}$$

According to the superposition principle, the mapping relationship between the output displacement ***U****_n_*_-4R_ of the mobile platform and the input forces ***F***_1_…***F***_j_ is as follows:

(31) $$U_{n\text{-}4R}=\left[\begin{array}{ccc} C_{F_{1}}^{O} & \cdots & C_{F_{j}}^{O} \end{array}\right]\left[\begin{array}{c} F_{1}\\ \vdots \\ F_{j} \end{array}\right]$$

where the non-redundant actuated mode corresponds to the case when the input forces other than ***F***_1_ and ***F***_2_ are 0.

So far, the kinetostatic model relating the input forces and the output displacements of the redundant actuated *n*-4R CPPM is established. The kinetostatic model between the input displacements and the output displacements is further established. It can be seen from Equation (15) that only the parameter ***K****_Bi_* is associated with the number of the mechanism’s branches. Therefore, the compensation equation of input force ***F****_i_* (*i* = 1, …, *j*) acting on the branch *i* to the equivalent input displacement $U_{F_{k}}$ (*k* = 1, …, *j*) acting on the branch *k* is as follows:

(32) $$\left[\begin{array}{c} F_{k}\\ F_{U}\\ F_{i} \end{array}\right]=\left[\begin{array}{ccc} K_{F_{k}F_{k}} & K_{F_{k}O} & 0\\ {Κ}_{OF_{k}} & {\left(K_{OO}\right)}_{F_{i}} & {Κ}_{OF_{i}}\\ 0 & K_{F_{i}O} & K_{F_{i}F_{i}} \end{array}\right]\left[\begin{array}{c} U_{F_{k}}\\ U_{k}\\ U_{F_{i}} \end{array}\right]\begin{array}{cc} & \left(i\neq k\right) \end{array}$$

where $U_{F_{k}}$ and $U_{F_{i}}$ represent the displacements at the force coordinate frame *F_k_xyz* and *F_i_xyz*, respectively. $U_{k}$ represents the displacement of the mobile platform in the global coordinate frame *Oxyz* resulting from force ***F****_k_*.

The stiffness matrices in Equation (32) are defined by:

(33) $$\left\{\begin{array}{c} {\left(K_{OO}\right)}_{F_{i}}=K_{BkB}^{O}+K_{BiB}^{O}+K_{n\text{-}4R}-K_{Bk}^{O}-K_{Bi}^{O}\\ K_{F_{i}F_{i}}=K_{BiA}^{F_{i}}+K_{BiB}^{F_{i}},K_{F_{k}F_{k}}=K_{BkA}^{F_{k}}+K_{BkB}^{F_{k}}\\ {Κ}_{OF_{i}}=-{\left(Ad_{O}^{O}\right)}^{-T}\left[K_{BiB}^{O}\right]{\left(Ad_{O}^{F_{i}}\right)}^{-1},K_{F_{i}O}=-{\left(Ad_{O}^{F_{i}}\right)}^{-T}\left[K_{BiB}^{O}\right]{\left(Ad_{O}^{O}\right)}^{-1}\\ {Κ}_{OF_{k}}=-{\left(Ad_{O}^{O}\right)}^{-T}\left[K_{BkB}^{O}\right]{\left(Ad_{O}^{F_{k}}\right)}^{-1},K_{F_{k}O}=-{\left(Ad_{O}^{F_{k}}\right)}^{-T}\left[K_{BkB}^{O}\right]{\left(Ad_{O}^{O}\right)}^{-1} \end{array}\right.$$

where:

(34) $$\left\{\begin{array}{c} K_{BiA}^{O}={\left(Ad_{2(i-1)\pi /n}\right)}^{-T}K_{B1A}^{O}{\left(Ad_{2(i-1)\pi /n}\right)}^{-1},K_{BiA}^{F_{i}}={\left(Ad_{O}^{F_{i}}\right)}^{-T}K_{BiA}^{O}{\left(Ad_{O}^{F_{i}}\right)}^{-1}\\ K_{BiB}^{O}={\left(Ad_{2(i-1)\pi /n}\right)}^{-T}K_{B1B}^{O}{\left(Ad_{2(i-1)\pi /n}\right)}^{-1},K_{BiB}^{F_{i}}={\left(Ad_{O}^{F_{i}}\right)}^{-T}K_{BiB}^{O}{\left(Ad_{O}^{F_{i}}\right)}^{-1}\\ K_{BkA}^{O}={\left(Ad_{2(k-1)\pi /n}\right)}^{-T}K_{B1A}^{O}{\left(Ad_{2(k-1)\pi /n}\right)}^{-1},K_{BiA}^{F_{k}}={\left(Ad_{O}^{F_{k}}\right)}^{-T}K_{BkA}^{O}{\left(Ad_{O}^{F_{k}}\right)}^{-1}\\ K_{BkB}^{O}={\left(Ad_{2(k-1)\pi /n}\right)}^{-T}K_{B1B}^{O}{\left(Ad_{2(k-1)\pi /n}\right)}^{-1},K_{BkB}^{F_{k}}={\left(Ad_{O}^{F_{k}}\right)}^{-T}K_{BkB}^{O}{\left(Ad_{O}^{F_{k}}\right)}^{-1}\\ K_{n\text{-}4R}={\left(C_{n\text{-}4R}\right)}^{-1},{\begin{array}{c} K_{Bk}^{O}={\left(Ad_{2(k-1)\pi /n}\right)}^{-T}\left(C_{1}\right) \end{array}}^{-1}{\left(Ad_{2(k-1)\pi /n}\right)}^{-1} \end{array}\right.$$

where ***C****_n_*_-4R_ denotes the total compliant matrix of *n*-4R CPPM, and its expression is presented in Formula (6) in literature [24].

From Equation (32), one can obtain:

(35) $$U_{F_{k}}=C_{F_{i}}^{F_{k}}F_{i}\begin{array}{cc} & \left(i\neq k\right) \end{array}$$

where $C_{F_{i}}^{F_{k}}={\left(K_{F_{k}F_{k}}-K_{F_{k}O}{\left(K_{R_{i}}\right)}^{-1}{Κ}_{OF_{k}}\right)}^{-1}\left(K_{F_{k}O}{\left(K_{R_{i}}\right)}^{-1}{Κ}_{OF_{i}}{\left(K_{F_{i}F_{i}}\right)}^{-1}\right)$, in which $K_{R_{i}}={\left(K_{OO}\right)}_{F_{i}}-{Κ}_{OF_{i}}{\left(K_{F_{i}F_{i}}\right)}^{-1}K_{F_{i}O}$. When solving the mapping matrix of input force and input displacement acting on the same branch, *i* = *k*, it can be obtained similarly to Equation (8):

(36) $$C_{F_{i}}^{F_{k}}={\left(K_{F_{i}F_{i}}-K_{F_{i}O}{\left(K_{OO}\right)}_{F_{i}}^{-1}K_{OF_{i}}\right)}^{-1}$$

According to the superposition principle, the total input displacement $U_{F_{k}\_total}$ at the force coordinate frame *F_k_xyz* can be obtained:

(37) $$U_{F_{k}\_total}=\left[\begin{array}{ccc} C_{F_{1}}^{F_{k}} & \cdots & C_{F_{j}}^{F_{k}} \end{array}\right]\left[\begin{array}{c} F_{1}\\ \vdots \\ F_{j} \end{array}\right]\begin{array}{cc} & \left(k=1,\ldots ,j\right) \end{array}$$

Then, the total input displacement $U_{F_{k}\_total}$ at the force coordinate frame *F_k_xyz* generated by the action of the j input forces ***F***_1_…***F****_j_* can be obtained by rearranging Equations (35) and (37), as shown in Equation (38).

(38) $$\left[\begin{array}{c} U_{F_{1}\_total}\\ \vdots \\ U_{F_{j}\_total} \end{array}\right]=\left[\begin{array}{ccc} C_{F_{1}}^{F_{1}} & \cdots & C_{F_{j}}^{F_{1}}\\ \vdots & \ddots & \vdots \\ C_{F_{1}}^{F_{j}} & \cdots & C_{F_{j}}^{F_{j}} \end{array}\right]\left[\begin{array}{c} F_{1}\\ \vdots \\ F_{j} \end{array}\right]$$

By substituting Equation (38) into (31), the relationship between the input displacements $U_{F_{k}\_total}$ at each force coordinate frame *F_k_xyz* and the output displacement ***U****_n_*_-4R_ of the mobile platform can be calculated as:

(39) $$U_{n\text{-}4R}=\left[\begin{array}{ccc} C_{F_{1}}^{O} & \cdots & C_{F_{j}}^{O} \end{array}\right]{\left[\begin{array}{ccc} C_{F_{1}}^{F_{1}} & \cdots & C_{F_{j}}^{F_{1}}\\ \vdots & \ddots & \vdots \\ C_{F_{1}}^{F_{j}} & \cdots & C_{F_{j}}^{F_{j}} \end{array}\right]}^{-1}\left[\begin{array}{c} U_{F_{1}\_total}\\ \vdots \\ U_{F_{j}\_total} \end{array}\right]$$

where the non-redundant actuated mode corresponds to the case when the input displacements other than $U_{F_{1}\_total}$ and $U_{F_{2}\_total}$ are 0.

A simplified kinetostatic model directly relating the output angular displacements in the direction of two degrees of freedom with j input component forces or displacements along the y-direction can be extracted from the kinetostatic model of the redundant actuated *n*-4R CPPM. Because the extraction results are similar to Equations (12) and (26), respectively, the double calculation is not provided here.

## 4. The Workspace of the Redundant Actuated *n*-4R CPPM

The commonly used method for calculating the workspace of a compliant mechanism is the pseudo-rigid-body model method. This method only takes into account the displacement of the hinge in the functional direction, and considers that there is no displacement in other degrees of freedom directions. Therefore, in essence, this method simplifies the compliant mechanism into a rigid mechanism. However, in order to better reflect the motion characteristics of the compliant mechanism and calculate the workspace of the compliant mechanism more accurately, it is necessary to consider the displacements generated by the flexible hinge in the six degrees of freedom directions during the motion of the mechanism. Therefore, the relationship relating the input forces/displacements and the flexible hinge displacement of the redundant actuated *n*-4R CPPM (hereinafter referred to as the flexible hinge displacement model) will be derived in this section.

### 4.1. The Constraints of the Workspace

Before proceeding with a workspace analysis, it is necessary to determine the maximum input force/displacement (*F_max_* and *d_max_*) of actuator and the maximum displacement of the flexible hinge, as these factors will limit the size, volume, and area shape characteristics of the workspace. Therefore, the constraints of the workspace are summarized as follows:

(40) $$\left\{\begin{array}{c} D_{ij}\leq U_{\max}\\ -F_{\max}\leq f_{i,y}\leq F_{\max}\\ -d_{\max}\leq d_{i}\leq d_{\max} \end{array}\right.$$

where:

(41) $$\left\{\begin{array}{c} D_{ij}={\left[\begin{array}{cccccc} \theta_{x} & \theta_{y} & \theta_{z} & \delta_{x} & \delta_{y} & \delta_{z} \end{array}\right]}^{T}\\ U_{\max}={\left[\begin{array}{cccccc} \theta_{x\max} & \theta_{y\max} & \theta_{z\max} & \delta_{x\max} & \delta_{y\max} & \delta_{z\max} \end{array}\right]}^{T} \end{array}\right.$$

where ***D****_ij_* represents the displacement of the flexible hinge R*_ij_* during the motion of the mechanism, and ***U****_max_* represents the maximum displacement of the flexible hinge. The subscripts *i* (*i* = 1, …, *n*) and *j* ( = 1, …, 4) in ***D****_ij_* indicate the number of branches and the number of flexible hinges corresponding to each branch, respectively.

The maximum displacement of the flexible hinge can be obtained by the FE-method. Due to the symmetry of the flexible hinge, one end face is fixed, and the load is gradually exerted on the center of the other end face. The displacement of the loaded end face is the displacement of the flexible hinge. Until the displacement of the flexible hinge no longer changes significantly with the increase of load, this usually indicates that the critical condition for the failure of the flexible hinge has been reached. Finally, the displacement corresponding to the loaded end face of the flexible hinge is obtained by the probe tool, and the displacement is the maximum displacement. Since this section is only a theoretical calculation, the maximum displacement of the flexible hinge does not have a safety factor, and if the motion control of the mechanism is required, an appropriate safety factor can be set to ensure the safety of the design.

### 4.2. The Flexible Hinge Displacement Model of the Redundant Actuated 3-4R CPPM

**Figure 4:**
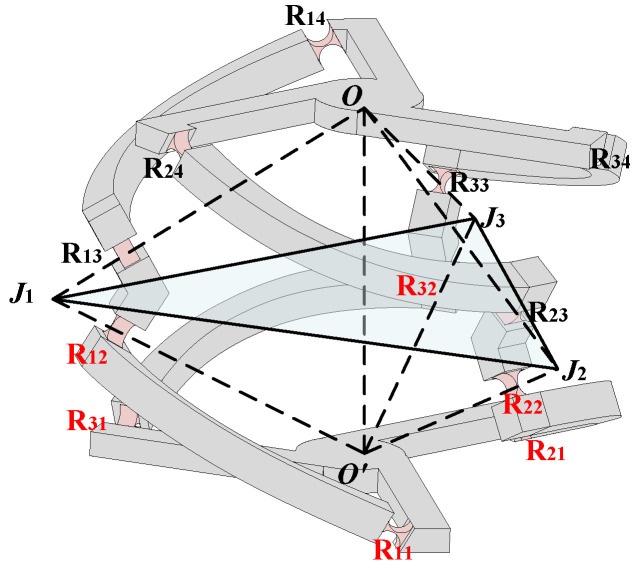
The symmetry of 3-4R CPPM.

Calculating the displacement of the flexible hinge is a difficult part of analyzing the workspace. In this section, the redundant actuated 3-4R CPPM is taken as an example to establish the mapping relationship between the input force/displacement of the mechanism and the displacement of the flexible hinge. As shown in Figure 4, due to the symmetry of the structure, there is always a symmetry plane that makes the motion of the upper and lower parts of the 3-4R CPPM symmetrical, so the deformation of the flexible hinge at the corresponding position of the upper and lower parts of the same branch is the same. The vertices of the symmetry plane are the intersection of the axes of the flexible hinge R*_i_*_2_ and R*_i_*_3_ (*i* = 1, 2, 3), and the intersection is defined as *J_i_*. Since the actuator acts on the connecting rod of each branch close to the fixed platform, six flexible hinges (R*_i_*_1_ and R*_i_*_2_) near the fixed platform are selected as the research objects, respectively.

The displacement of flexible hinge can be regarded as the superposition of the displacements generated by each individual input force/displacement acting separately. Take branch 1 as an example, consider that the mechanism is only subjected to the force ***F****_U_* in the center *O* of the mobile platform. First, the displacement of the flexible hinge R_11_ directly connected to the fixed platform is analyzed. One end of the hinge is fixed on the fixed platform, and the other end has the corresponding displacement during the motion of the mechanism. So, the displacement of the hinge coordinate frame *O*_11_*xyz* is the displacement of the hinge. Therefore, to describe the displacement ***U***_11_ of flexible hinge R_11_ resulting from force ***F****_U_* in the terms of compliance matrix, Equation (42) yields:

(42) $$U_{11}=C_{O}^{O_{11}}\cdot F_{U}$$

where $C_{O}^{O_{11}}$ is the mapping matrix between force ***F****_U_* and displacement ***U***_11_. The generalized displacement is defined as: ***U***_11_ = [*θ_x_*, *θ_y_*, *θ_z_*, *δ_x_*, *δ_y_*, *δ_z_*]^T^.

Since the point *O*_11_ and the point *F*_1_ are located on the same rod, $C_{O}^{O_{11}}$ can be obtained directly by coordinate transformation.

(43) $$C_{O}^{O_{11}}=Ad_{F_{1}}^{O_{11}}\cdot C_{O}^{F_{1}},Ad_{F_{1}}^{O_{11}}=Ad_{O}^{O_{11}}\cdot Ad_{F_{1}}^{O},Ad_{F_{1}}^{O}={\left(Ad_{O}^{F_{1}}\right)}^{-1}$$

where matrix $Ad_{O}^{O_{11}}$ represents the coordinate transformation from global coordinate frame *Oxyz* to coordinate frame *O*_11_*xyz*, which can be computed by:

(44) $$\begin{array}{l} Ad_{O}^{O_{1i}}={\left(Ad_{O_{1i}}^{O}\right)}^{-1},\begin{array}{c} Ad_{O_{1i}}^{O}=\left[\begin{array}{cc} R_{O_{1i}}^{O} & 0\\ T_{O_{1i}}^{O}\cdot R_{O_{1i}}^{O} & R_{O_{1i}}^{O} \end{array}\right] \end{array},\\ T_{O_{1i}}^{O}=\left[\begin{array}{ccc} 0 & -z & y\\ z & 0 & -x\\ -y & x & 0 \end{array}\right],R_{O_{1i}}^{O}=\left[\begin{array}{ccc} c\beta c\gamma & -c\beta s\gamma & s\beta \\ c\alpha s\gamma +c\gamma s\alpha s\beta & c\alpha c\gamma -s\alpha s\beta s\gamma & -c\beta s\alpha \\ s\alpha s\gamma -c\alpha c\gamma s\beta & c\gamma s\alpha +c\alpha s\beta s\gamma & c\alpha c\beta \end{array}\right] \end{array}$$

where $T_{O_{1i}}^{O}$ (*i* = 1, 2) is the antisymmetric matrix defined by the translation vector $t_{O_{1i}}^{O}={\left(x,y,z\right)}^{T}$. $R_{O_{1i}}^{O}$ is the rotation transformation matrix of coordinate frame *O*_1*i*_*xyz* to the global coordinate frame *Oxyz*. *α*, *β* and *γ* are the angle of rotation around *x*, *y* and *z* axis of coordinate frame *O*_1*i*_*xyz*, respectively. s and c respectively represent sin and cos. The parameters are listed in Table 1.

Since two ends of the flexible hinge R_12_ are free ends, the displacement of the hinge is composed of two parts, which are the displacements of the hinge coordinate frames *O*_12A_*xyz* and *O*_12B_*xyz*. Since the point *O*_12A_ and the point *F*_1_ are located on the same rod, the mapping matrix $C_{O}^{O_{12A}}$ between force ***F****_U_* and the displacement ***U***_12A_ of coordinate frame *O*_12A_*xyz* can also be obtained directly by the coordinate transformation.

(45) $$C_{O}^{O_{12A}}=Ad_{F_{1}}^{O_{12A}}\cdot C_{O}^{F_{1}}$$

where matrix $Ad_{F_{1}}^{O_{12A}}$ represents the coordinate transformation from force coordinate frame *F*_1_*xyz* to coordinate frame *O*_12A_*xyz*, which can be computed by:

(46) $$Ad_{F_{1}}^{O_{12A}}=Ad_{O_{12B}}^{O_{12A}}\cdot Ad_{O}^{O_{12B}}\cdot {\left(Ad_{O}^{F_{1}}\right)}^{-1}$$

where matrix $Ad_{O}^{O_{12B}}$ represents the coordinate transformation from global coordinate frame *Oxyz* to coordinate frame *O*_12B_*xy*, and $Ad_{O}^{O_{12B}}={\left(Ad_{O_{12}}^{O}\right)}^{-1}$. Matrix $Ad_{O_{12B}}^{O_{12A}}$ represents the coordinate transformation from coordinate frame *O*_12B_*xyz* to coordinate frame *O*_12A_*xyz*, which can be computed by:

(47) $$Ad_{O_{12B}}^{O_{12A}}=\left[\begin{array}{cc} I_{3\times 3} & 0_{3\times 3}\\ T_{O_{12B}}^{O_{12A}} & I_{3\times 3} \end{array}\right],T_{O_{12B}}^{O_{12A}}=\left[\begin{array}{ccc} 0 & 0 & 0\\ 0 & 0 & -2r\\ 0 & 2r & 0 \end{array}\right]$$

where $T_{O_{12B}}^{O_{12A}}$ is the antisymmetric matrix defined by the translation vector $t_{O_{12B}}^{O_{12A}}={\left(2r,0,0\right)}^{T}$, and *r* denotes the cutting radius of the flexible hinge.

The calculation method of the mapping matrix of force ***F****_U_* and coordinate frame *O*_12B_*xyz* displacement ***U***_12B_ is similar to that of mechanism kinetostatic modeling. To avoid repetition, conclusions are given directly:

(48) $$C_{O}^{O_{12B}}=-{\left({\left(K_{OO}\right)}_{F_{U}}-K_{OF_{U}}K_{F_{U}F_{U}}^{-1}K_{F_{U}O}\right)}^{-1}\left(K_{OF_{U}}K_{F_{U}F_{U}}^{-1}\right)$$

where:

(49) $$\left\{\begin{array}{c} K_{F_{U}F_{U}}=K_{B1B}^{O}+K_{B2}^{O}+K_{B3}^{O},{\left(K_{OO}\right)}_{F_{U}}=K_{B1A}^{O_{12B}}+K_{B1B}^{O_{12B}}\\ {Κ}_{OF_{U}}=-{\left(Ad_{O_{12}}^{O}\right)}^{T}\left[K_{B1B}^{O}\right]\left(Ad_{O}^{O}\right),K_{F_{U}O}=-{\left(Ad_{O}^{O}\right)}^{T}\left[K_{B1B}^{O}\right]\left(Ad_{O_{12}}^{O}\right) \end{array}\right.$$

where:

(50) $$\left\{\begin{array}{c} K_{B1A}^{O}={\left(C_{11}+C_{12}\right)}^{-1},K_{B1B}^{O}={\left(C_{13}+C_{14}\right)}^{-1}\\ {\begin{array}{c} K_{B2}^{O}=\left(C_{2}\right) \end{array}}^{-1},{\begin{array}{c} K_{B3}^{O}=\left(C_{3}\right) \end{array}}^{-1}\\ K_{B1A}^{O_{12B}}={\left({\left(Ad_{O_{12}}^{O}\right)}^{-1}\cdot {\left(K_{B1A}^{O}\right)}^{-1}\cdot {\left(Ad_{O_{12}}^{O}\right)}^{-T}\right)}^{-1}\\ K_{B1B}^{O_{12B}}={\left({\left(Ad_{O_{12}}^{O}\right)}^{-1}\cdot {\left(K_{B1B}^{O}\right)}^{-1}\cdot {\left(Ad_{O_{12}}^{O}\right)}^{-T}\right)}^{-1} \end{array}\right.$$

Since the three branches of the 3-4R CPPM are uniform and evenly distributed in circumferential directions at 120° intervals, the mapping matrices of the force ***F****_U_* and the displacements of flexible hinges R_i1_ and R_i2_ (*i* = 2, 3) on the remaining branches can be acquired by rotating the compliance matrices $C_{O}^{O_{11}}$,$C_{O}^{O_{12A}}$ and $C_{O}^{O_{12B}}$ by 120° and 240° around the *y*-axis of the global coordinate frame *Oxyz*, respectively.

(51) $$\left\{\begin{array}{c} C_{O}^{O_{i1}}\;\;=C_{O}^{O_{11}}\cdot {\left(Ad_{2(i-1)\pi /3}\right)}^{T}\\ C_{O}^{O_{i2A}}=C_{O}^{O_{12A}}\cdot {\left(Ad_{2(i-1)\pi /3}\right)}^{T}\\ C_{O}^{O_{i2B}}=C_{O}^{O_{12B}}\cdot {\left(Ad_{2(i-1)\pi /3}\right)}^{T} \end{array}\right.$$

So far, the mapping relationship of the force ***F****_U_* and the displacements of flexible hinges R*_i_*_1_ and R*_i_*_2_ (*i* = 1, 2, 3) is obtained. Assuming the mechanism is only affected by the input force ***F***_1_ exerted on the branch 1, the mapping relationship between the force ***F***_1_ and the displacements of the flexible hinges R_11_ and R_12_ is further obtained, as shown in Equation (52).

(52) $$U_{11}=C_{F_{1}}^{O_{11}}\cdot F_{1},U_{12A}=C_{F_{1}}^{O_{12A}}\cdot F_{1},U_{12B}=C_{F_{1}}^{O_{12B}}\cdot F_{1}$$

where $C_{F_{1}}^{O_{11}}$, $C_{F_{1}}^{O_{12A}}$ and $C_{F_{1}}^{O_{12B}}$ are the mapping matrices between force ***F***_1_ and displacement ***U***_11_, ***U***_12A_, and ***U***_12B_.

The points *O*_11_ and *O*_12A_ are both on the same rod member as the point *F*_1_, so the matrices $C_{F_{1}}^{O_{11}}$ and $C_{F_{1}}^{O_{12A}}$ can be respectively obtained from the matrix $C_{F_{1}}^{F_{1}}$ in Equation (19) by the coordinate transformation.

(53) $$C_{F_{1}}^{O_{11}}=Ad_{F_{1}}^{O_{11}}\cdot C_{F_{1}}^{F_{1}},C_{F_{1}}^{O_{12A}}=Ad_{F_{1}}^{O_{12A}}\cdot C_{F_{1}}^{F_{1}}$$

Matrix $C_{F_{1}}^{O_{12B}}$ can still be obtained using kinetostatic modeling methods.

(54) $$C_{F_{1}}^{O_{12B}}=-{\left({\left(K_{OO}\right)}_{F_{1}}-K_{OF_{1}}K_{F_{1}F_{1}}^{-1}K_{F_{1}O}\right)}^{-1}\left(K_{OF_{1}}K_{F_{1}F_{1}}^{-1}\right)$$

where:

(55) $$\left\{\begin{array}{c} {\left(K_{OO}\right)}_{F_{1}}={\left({\left(K_{B2}^{O_{12B}}+K_{B3}^{O_{12B}}\right)}^{-1}+C_{13}^{O_{12B}}+C_{14}^{O_{12B}}\right)}^{-1}+{\left(C_{12}^{O_{12B}}\right)}^{-1}\\ K_{F_{1}F_{1}}={\left(C_{11}^{F_{1}}\right)}^{-1}+{\left(C_{12}^{F_{1}}\right)}^{-1}\\ {Κ}_{OF_{1}}=-{\left(Ad_{O_{12}}^{O}\right)}^{T}{\left[C_{12}\right]}^{-1}{\left(Ad_{O}^{F_{1}}\right)}^{-1}\\ K_{F_{1}O}=-{\left(Ad_{O}^{F_{1}}\right)}^{-T}{\left[C_{12}\right]}^{-1}\left(Ad_{O_{12}}^{O}\right) \end{array}\right.$$

where:

(56) $$\left\{\begin{array}{c} K_{B2}^{O_{12B}}={\left(C_{B2}^{O_{12B}}\right)}^{-1},C_{B2}^{O_{12B}}=Ad_{O}^{O_{12B}}\left(C_{2}\right){\left(Ad_{O}^{O_{12B}}\right)}^{T}\\ K_{B3}^{O_{12B}}={\left(C_{B3}^{O_{12B}}\right)}^{-1},C_{B3}^{O_{12B}}=Ad_{O}^{O_{12B}}\left(C_{3}\right){\left(Ad_{O}^{O_{12B}}\right)}^{T}\\ C_{12}^{O_{12B}}=Ad_{O}^{O_{12B}}\left(C_{12}\right){\left(Ad_{O}^{O_{12B}}\right)}^{T},C_{13}^{O_{12B}}=Ad_{O}^{O_{12B}}\left(C_{13}\right){\left(Ad_{O}^{O_{12B}}\right)}^{T}\\ C_{14}^{O_{12B}}=Ad_{O}^{O_{12B}}\left(C_{14}\right){\left(Ad_{O}^{O_{12B}}\right)}^{T}\\ C_{11}^{F_{1}}=Ad_{O}^{F_{1}}\cdot C_{11}\cdot {\left(Ad_{O}^{F_{1}}\right)}^{T},C_{12}^{F_{1}}=Ad_{O}^{F_{1}}\cdot C_{12}\cdot {\left(Ad_{O}^{F_{1}}\right)}^{T} \end{array}\right.$$

From the obtained matrices $C_{F_{1}}^{O}$, $C_{O}^{O_{i1}}$, $C_{O}^{O_{i2A}}$, $C_{O}^{O_{i2B}}$, and the compliance matrix ***C***_3-4R_, the mapping relationship between the force ***F***_1_ and the displacements of the flexible hinges R*_i_*_1_ and R*_i_*_2_ (*i* = 2, 3) on the remaining branches can be obtained.

(57) $$\left\{\begin{array}{c} U_{i1}=C_{F_{1}}^{O_{i1}}\cdot F_{1}\;\;,\;\;\;\;C_{F_{1}}^{O_{i1}}\;\;=C_{O}^{O_{i1}}\cdot {\left(C_{3\text{-}4R}\right)}^{-1}\cdot C_{F_{1}}^{O}\\ U_{i2A}=C_{F_{1}}^{O_{i2A}}\cdot F_{1}\;,\;\;\;\;C_{F_{1}}^{O_{i2A}}=C_{O}^{O_{i2A}}\cdot {\left(C_{3\text{-}4R}\right)}^{-1}\cdot C_{F_{1}}^{O}\\ U_{i2B}=C_{F_{1}}^{O_{i2B}}\cdot F_{1}\;,\;\;\;\;C_{F_{1}}^{O_{i2B}}=C_{O}^{O_{i2B}}\cdot {\left(C_{3\text{-}4R}\right)}^{-1}\cdot C_{F_{1}}^{O} \end{array}\right.$$

So far, the mapping relationship of the force ***F***_1_ and the displacements of flexible hinges R*_i_*_1_ and R*_i_*_2_ (*i* = 1, 2, 3) is obtained. Due to the symmetry of the structure, the mapping matrices of the forces ***F***_2_ and ***F***_3_ and the displacements of flexible hinges R*_i_*_1_ and R*_i_*_2_ can be directly obtained using the results of the compliance matrices $C_{F_{1}}^{O_{i1}}$, $C_{F_{1}}^{O_{i2A}}$ and $C_{F_{1}}^{O_{i2B}}$, as shown in Equation (58).

(58) $$\left\{\begin{array}{c} C_{F_{2}}^{O_{11}}=C_{F_{1}}^{O_{31}},C_{F_{2}}^{O_{21}}=C_{F_{1}}^{O_{11}},C_{F_{2}}^{O_{31}}=C_{F_{1}}^{O_{21}}\\ C_{F_{2}}^{O_{12A}}=C_{F_{1}}^{O_{32A}},C_{F_{2}}^{O_{22A}}=C_{F_{1}}^{O_{12A}},C_{F_{2}}^{O_{32A}}=C_{F_{1}}^{O_{22A}}\\ C_{F_{2}}^{O_{12B}}=C_{F_{1}}^{O_{32B}},C_{F_{2}}^{O_{22B}}=C_{F_{1}}^{O_{12B}},C_{F_{2}}^{O_{32B}}=C_{F_{1}}^{O_{22B}}\\ C_{F_{3}}^{O_{11}}=C_{F_{1}}^{O_{21}},C_{F_{3}}^{O_{21}}=C_{F_{1}}^{O_{31}},C_{F_{3}}^{O_{31}}=C_{F_{1}}^{O_{11}}\\ C_{F_{3}}^{O_{12A}}=C_{F_{1}}^{O_{22A}},C_{F_{3}}^{O_{22A}}=C_{F_{1}}^{O_{32A}},C_{F_{3}}^{O_{32A}}=C_{F_{1}}^{O_{12A}}\\ C_{F_{3}}^{O_{12B}}=C_{F_{1}}^{O_{22B}},C_{F_{3}}^{O_{22B}}=C_{F_{1}}^{O_{32B}},C_{F_{3}}^{O_{32B}}=C_{F_{1}}^{O_{12B}} \end{array}\right.$$

The total displacements of flexible hinges R*_i_*_1_ and R*_i_*_2_ can be regarded as the superposition of the displacements caused by the input forces ***F***_1_, ***F***_2_ and ***F***_3_ acting alone, one can obtained:

(59) $$\left[\begin{array}{c} {\left(U_{i1}\right)}_{total}\\ {\left(U_{i2A}\right)}_{total}\\ {\left(U_{i2B}\right)}_{total} \end{array}\right]=\left[\begin{array}{ccc} C_{F_{1}}^{O_{i1}} & C_{F_{2}}^{O_{i1}} & C_{F_{3}}^{O_{i1}}\\ C_{F_{1}}^{O_{i2A}} & C_{F_{2}}^{O_{i2A}} & C_{F_{3}}^{O_{i2A}}\\ C_{F_{1}}^{O_{i2B}} & C_{F_{2}}^{O_{i2B}} & C_{F_{3}}^{O_{i2B}} \end{array}\right]\left[\begin{array}{c} F_{1}\\ F_{2}\\ F_{3} \end{array}\right]\begin{array}{cc} & \left(i=1,2,3\right) \end{array}$$

where ${\left(U_{i1}\right)}_{total}$, ${\left(U_{i2A}\right)}_{total}$ and ${\left(U_{i2B}\right)}_{total}$ represent the total displacements of the hinge coordinate frames *O*_11_*xyz*, *O*_12A_*xyz*, and *O*_12B_*xyz*, respectively.

By substituting Equation (23) into (59), the relationship between the input displacements and the displacements of flexible hinges can be calculated as:

(60) $$\left[\begin{array}{c} {\left(U_{i1}\right)}_{total}\\ {\left(U_{i2A}\right)}_{total}\\ {\left(U_{i2B}\right)}_{total} \end{array}\right]=\left[\begin{array}{ccc} C_{F_{1}}^{O_{i1}} & C_{F_{2}}^{O_{i1}} & C_{F_{3}}^{O_{i1}}\\ C_{F_{1}}^{O_{i2A}} & C_{F_{2}}^{O_{i2A}} & C_{F_{3}}^{O_{i2A}}\\ C_{F_{1}}^{O_{i2B}} & C_{F_{2}}^{O_{i2B}} & C_{F_{3}}^{O_{i2B}} \end{array}\right]{\left[\begin{array}{ccc} C_{F_{1}}^{F_{1}} & C_{F_{2}}^{F_{1}} & C_{F_{3}}^{F_{1}}\\ C_{F_{1}}^{F_{2}} & C_{F_{2}}^{F_{2}} & C_{F_{3}}^{F_{2}}\\ C_{F_{1}}^{F_{3}} & C_{F_{2}}^{F_{3}} & C_{F_{3}}^{F_{3}} \end{array}\right]}^{-1}\left[\begin{array}{c} U_{F_{1}\_total}\\ U_{F_{2}\_total}\\ U_{F_{3}\_total} \end{array}\right]$$

It should be noted that, when the input force ***F****_i_* = [0, 0, 0, 0, *f*_*i*,*y*_, 0]^T^ and the input displacement $U_{F_{i}\_total}$ = [0, 0, 0, 0, *d_i_*, 0]^T^ in Equations (59) and (60), the mapping relationship between the input force/displacement along the *y*-direction and the displacement of each flexible hinge can be obtained. Since the calculation process is similar to that of the kinetostatic model, the calculation will not be repeated here.

### 4.3. The Flexible Hinge Displacement Model of the Redundant Actuated n-4R CPPM

In this section, the flexible hinge displacement calculation model is further extended from redundant actuated 3-4R to the redundant actuated *n*-4R CPPM. It can be seen from Equations (3), (50), and (55) that only the parameters ***K****_B_*_2_ and ***K****_B_*_3_ are associated with the number of the mechanism’s branches. Therefore, in order to avoid repetition, this section directly gives relevant conclusions. The mapping matrix between force ***F****_U_* exerted on the mobile platform center and the displacement ***U***_11_ of flexible hinge R_11_ is given directly:

(61) $$C_{O}^{O_{11}}=Ad_{F_{1}}^{O_{11}}\cdot C_{O}^{F_{1}},C_{O}^{F_{1}}=-{\left(K_{F_{1}F_{1}}-K_{F_{1}O}{\left(K_{OO}\right)}_{F_{1}}^{-1}K_{OF_{1}}\right)}^{-1}K_{F_{1}O}{\left(K_{OO}\right)}_{F_{1}}^{-1}$$

where stiffness matrices can be obtained from Equation (29).

The mapping matrix between force ***F****_U_* and the displacement ***U***_12A_ of coordinate frame *O*_12A_*xyz* is as follows:

(62) $$C_{O}^{O_{12A}}=Ad_{F_{1}}^{O_{12A}}\cdot C_{O}^{F_{1}}$$

The mapping matrix between force ***F****_U_* and the displacement ***U***_12B_ of coordinate frame *O*_12B_*xyz* is as follows:

(63) $$C_{O}^{O_{12B}}=-{\left({\left(K_{OO}\right)}_{F_{U}}-K_{OF_{U}}K_{F_{U}F_{U}}^{-1}K_{F_{U}O}\right)}^{-1}\left(K_{OF_{U}}K_{F_{U}F_{U}}^{-1}\right)$$

where:

(64) $$\left\{\begin{array}{c} K_{F_{U}F_{U}}=K_{B1B}^{O}+K_{n\text{-}4R}-K_{B1}^{O},{\left(K_{OO}\right)}_{F_{U}}=K_{B1A}^{O_{12B}}+K_{B1B}^{O_{12B}}\\ {Κ}_{OF_{U}}=-{\left(Ad_{O_{12}}^{O}\right)}^{T}\left[K_{B1B}^{O}\right]\left(Ad_{O}^{O}\right),K_{F_{U}O}=-{\left(Ad_{O}^{O}\right)}^{T}\left[K_{B1B}^{O}\right]\left(Ad_{O_{12}}^{O}\right) \end{array}\right.$$

where:

(65) $$\left\{\begin{array}{c} K_{B1A}^{O}={\left(C_{11}+C_{12}\right)}^{-1},K_{B1A}^{O_{12B}}={\left({\left(Ad_{O_{12}}^{O}\right)}^{-1}\cdot {\left(K_{B1A}^{O}\right)}^{-1}\cdot {\left(Ad_{O_{12}}^{O}\right)}^{-T}\right)}^{-1}\\ K_{B1B}^{O}={\left(C_{13}+C_{14}\right)}^{-1},K_{B1B}^{O_{12B}}={\left({\left(Ad_{O_{12}}^{O}\right)}^{-1}\cdot {\left(K_{B1B}^{O}\right)}^{-1}\cdot {\left(Ad_{O_{12}}^{O}\right)}^{-T}\right)}^{-1}\\ {\begin{array}{c} K_{n\text{-}4R}={\left(C_{n\text{-}4R}\right)}^{-1},K_{B1}^{O}=\left(C_{1}\right) \end{array}}^{-1} \end{array}\right.$$

Since the *n* branches of the *n*-4R CPPM are uniform and evenly distributed in circumferential directions at 2π/*n* intervals, the mapping matrices of the force ***F****_U_* and the displacements of flexible hinges and R*_i_*_2_ (*i* = 2, …, *n*) on the remaining branches can be acquired by rotating the compliance matrices $C_{O}^{O_{11}}$, $C_{O}^{O_{12A}}$ and $C_{O}^{O_{12B}}$ by 2 (*i* − 1)π/*n* around the *y*-axis of the global coordinate frame *Oxyz*, respectively.

(66) $$\left\{\begin{array}{c} C_{O}^{O_{i1}}\;\;=C_{O}^{O_{11}}\cdot {\left(Ad_{2(i-1)\pi /n}\right)}^{T}\\ C_{O}^{O_{i2A}}=C_{O}^{O_{12A}}\cdot {\left(Ad_{2(i-1)\pi /n}\right)}^{T}\\ C_{O}^{O_{i2B}}=C_{O}^{O_{12B}}\cdot {\left(Ad_{2(i-1)\pi /n}\right)}^{T} \end{array}\right.$$

Similarly, assuming the mechanism is only affected by the input force ***F***_1_ exerted on branch 1, the matrices $C_{F_{1}}^{O_{11}}$ and $C_{F_{1}}^{O_{12A}}$ can be respectively obtained from the matrix $C_{F_{1}}^{F_{1}}$ in Equation (36) by the coordinate transformation.

(67) $$C_{F_{1}}^{O_{11}}=Ad_{F_{1}}^{O_{11}}\cdot C_{F_{1}}^{F_{1}},C_{F_{1}}^{O_{12A}}=Ad_{F_{1}}^{O_{12A}}\cdot C_{F_{1}}^{F_{1}}$$

Matrix $C_{F_{1}}^{O_{12B}}$ can be obtained using kinetostatic modeling methods.

(68) $$C_{F_{1}}^{O_{12B}}=-{\left({\left(K_{OO}\right)}_{F_{1}}-K_{OF_{1}}K_{F_{1}F_{1}}^{-1}K_{F_{1}O}\right)}^{-1}\left(K_{OF_{1}}K_{F_{1}F_{1}}^{-1}\right)$$

where:

(69) $$\left\{\begin{array}{c} {\left(K_{OO}\right)}_{F_{1}}={\left({\left(K_{n\text{-}4R}^{O_{12B}}-K_{B1}^{O_{12B}}\right)}^{-1}+C_{13}^{O_{12B}}+C_{14}^{O_{12B}}\right)}^{-1}+{\left(C_{12}^{O_{12B}}\right)}^{-1}\\ K_{F_{1}F_{1}}={\left(C_{11}^{F_{1}}\right)}^{-1}+{\left(C_{12}^{F_{1}}\right)}^{-1}\\ {Κ}_{OF_{1}}=-{\left(Ad_{O_{12}}^{O}\right)}^{T}{\left[C_{12}\right]}^{-1}{\left(Ad_{O}^{F_{1}}\right)}^{-1}\\ K_{F_{1}O}=-{\left(Ad_{O}^{F_{1}}\right)}^{-T}{\left[C_{12}\right]}^{-1}\left(Ad_{O_{12}}^{O}\right) \end{array}\right.$$

where:

(70) $$\left\{\begin{array}{c} K_{n\text{-}4R}^{O_{12B}}={\left(C_{n-4R}^{O_{12B}}\right)}^{-1},C_{n\text{-}4R}^{O_{12B}}=Ad_{O}^{O_{12B}}\left(C_{n\text{-}4R}\right){\left(Ad_{O}^{O_{12B}}\right)}^{T}\\ K_{B1}^{O_{12B}}={\left(C_{B1}^{O_{12B}}\right)}^{-1},C_{B1}^{O_{12B}}=Ad_{O}^{O_{12B}}\left(C_{1}\right){\left(Ad_{O}^{O_{12B}}\right)}^{T}\\ C_{12}^{O_{12B}}=Ad_{O}^{O_{12B}}\left(C_{12}\right){\left(Ad_{O}^{O_{12B}}\right)}^{T},C_{13}^{O_{12B}}=Ad_{O}^{O_{12B}}\left(C_{13}\right){\left(Ad_{O}^{O_{12B}}\right)}^{T}\\ C_{14}^{O_{12B}}=Ad_{O}^{O_{12B}}\left(C_{14}\right){\left(Ad_{O}^{O_{12B}}\right)}^{T}\\ C_{11}^{F_{1}}=Ad_{O}^{F_{1}}\cdot C_{11}\cdot {\left(Ad_{O}^{F_{1}}\right)}^{T},C_{12}^{F_{1}}=Ad_{O}^{F_{1}}\cdot C_{12}\cdot {\left(Ad_{O}^{F_{1}}\right)}^{T} \end{array}\right.$$

From the obtained matrices $C_{F_{1}}^{O}$, $C_{O}^{O_{i1}}$, $C_{O}^{O_{i2A}}$, $C_{O}^{O_{i2B}}$ and the compliance matrix ***C****_n_*_-4R_, the mapping relationship between the force ***F***_1_ and the displacements of the flexible hinges R*_i_*_1_ and R*_i_*_2_ (*i* = 2, …, *n*) can be obtained:

(71) $$\left\{\begin{array}{c} U_{i1}=C_{F_{1}}^{O_{i1}}\cdot F_{1}\;\;,\;\;\;\;C_{F_{1}}^{O_{i1}}\;\;=C_{O}^{O_{i1}}\cdot {\left(C_{n\text{-}4R}\right)}^{-1}\cdot C_{F_{1}}^{O}\\ U_{i2A}=C_{F_{1}}^{O_{i2A}}\cdot F_{1}\;,\;\;\;\;C_{F_{1}}^{O_{i2A}}=C_{O}^{O_{i2A}}\cdot {\left(C_{n\text{-}4R}\right)}^{-1}\cdot C_{F_{1}}^{O}\\ U_{i2B}=C_{F_{1}}^{O_{i2B}}\cdot F_{1}\;,\;\;\;\;C_{F_{1}}^{O_{i2B}}=C_{O}^{O_{i2B}}\cdot {\left(C_{n\text{-}4R}\right)}^{-1}\cdot C_{F_{1}}^{O} \end{array}\right.$$

Due to the symmetry of the structure, the mapping matrices of the force ***F**_k_* (*k* = 2, …, *j*) and the displacements of flexible hinges R*_i_*_1_ and R*_i_*_2_ (*i* = 1, …, *n*) can be directly obtained using the results of the compliance matrices $C_{F_{1}}^{O_{i1}}$, $C_{F_{1}}^{O_{i2A}}$ and $C_{F_{1}}^{O_{i2B}}$, as shown in Equation (72).

(72) $$\left\{\begin{array}{l} C_{F_{k}}^{O_{i1}}=C_{F_{1}}^{O_{\left(n-k+i+1\right)1}},C_{F_{k}}^{O_{i2A}}=C_{F_{1}}^{O_{\left(n-k+i+1\right)2A}},C_{F_{k}}^{O_{i2B}}=C_{F_{1}}^{O_{\left(n-k+i+1\right)2B}}\begin{array}{cc} & \left(i<k\right) \end{array}\\ C_{F_{k}}^{O_{i1}}=C_{F_{1}}^{O_{11}},C_{F_{k}}^{O_{i2A}}=C_{F_{1}}^{O_{12A}},C_{F_{k}}^{O_{i2B}}=C_{F_{1}}^{O_{12B}}\begin{array}{cc} & \left(i=k\right) \end{array}\\ C_{F_{k}}^{O_{i1}}=C_{F_{1}}^{O_{\left(i-k+1\right)1}},C_{F_{k}}^{O_{i2A}}=C_{F_{1}}^{O_{\left(i-k+1\right)2A}},C_{F_{k}}^{O_{i2B}}=C_{F_{1}}^{O_{\left(i-k+1\right)2B}}\begin{array}{cc} & \left(i>k\right) \end{array} \end{array}\right.$$

According to the superposition principle, the total displacements of flexible hinges R*_i_*_1_ and R*_i_*_2_ can be obtained:

(73) $$\left[\begin{array}{c} {\left(U_{i1}\right)}_{total}\\ {\left(U_{i2A}\right)}_{total}\\ {\left(U_{i2B}\right)}_{total} \end{array}\right]=\left[\begin{array}{ccc} C_{F_{1}}^{O_{i1}} & \cdots & C_{F_{j}}^{O_{i1}}\\ C_{F_{1}}^{O_{i2A}} & \cdots & C_{F_{j}}^{O_{i2A}}\\ C_{F_{1}}^{O_{i2B}} & \cdots & C_{F_{j}}^{O_{i2B}} \end{array}\right]\left[\begin{array}{c} F_{1}\\ \vdots \\ F_{j} \end{array}\right]$$

By substituting Equation (38) into (73), the relationship between the input displacements $U_{F_{k}\_total}$ (*k* = 1, …, j) and the displacements of flexible hinges can be calculated as:

(74) $$\left[\begin{array}{c} {\left(U_{i1}\right)}_{total}\\ {\left(U_{i2A}\right)}_{total}\\ {\left(U_{i2B}\right)}_{total} \end{array}\right]=\left[\begin{array}{ccc} C_{F_{1}}^{O_{i1}} & \cdots & C_{F_{j}}^{O_{i1}}\\ C_{F_{1}}^{O_{i2A}} & \cdots & C_{F_{j}}^{O_{i2A}}\\ C_{F_{1}}^{O_{i2B}} & \cdots & C_{F_{j}}^{O_{i2B}} \end{array}\right]{\left[\begin{array}{ccc} C_{F_{1}}^{F_{1}} & \cdots & C_{F_{j}}^{F_{1}}\\ \vdots & \ddots & \vdots \\ C_{F_{1}}^{F_{j}} & \cdots & C_{F_{j}}^{F_{j}} \end{array}\right]}^{-1}\left[\begin{array}{c} U_{F_{1}\_total}\\ \vdots \\ U_{F_{j}\_total} \end{array}\right]$$

## 5. Validation, Calculation and Analysis

### 5.1. Validation of the Kinetostatic Model

In this section, the correctness of the kinetostatic model of the redundant actuated 3-4R CPPM is verified through the comparison between the analytical calculation and FE-simulation using a given spatial pointing trajectory. The validation of the analytical results in the kinetostatic model of the redundant actuated 3-4R CPPM is provided by commercial software ANSYS 2022. Among them, a tetrahedral mesh with an element size of 2 mm is created for the links, fixed platform, and mobile platform, and mesh refinements with an element size of 0.2 mm are performed at the right-circular flexure hinges. The structural parameters of the mechanism are shown in Table 2.

**Figure 5:**
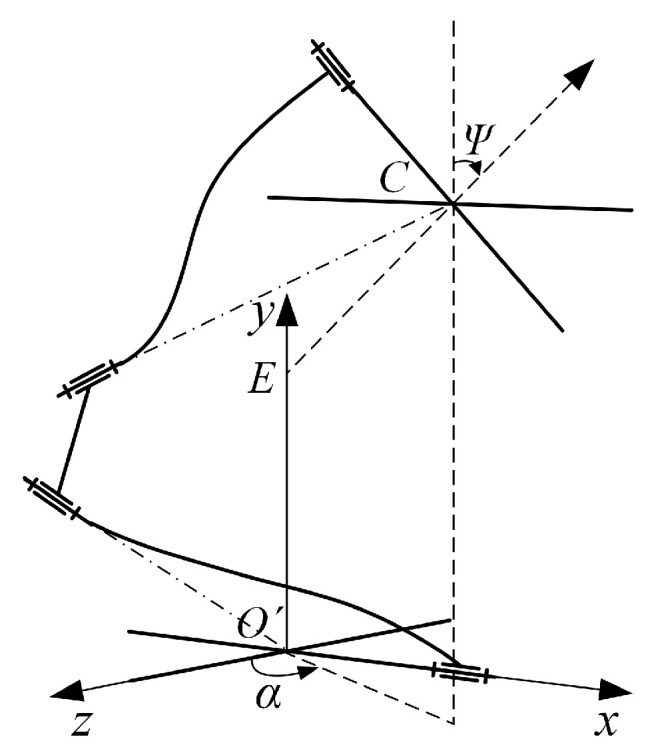
Spatial pointing of mechanism.

**Figure 6:**
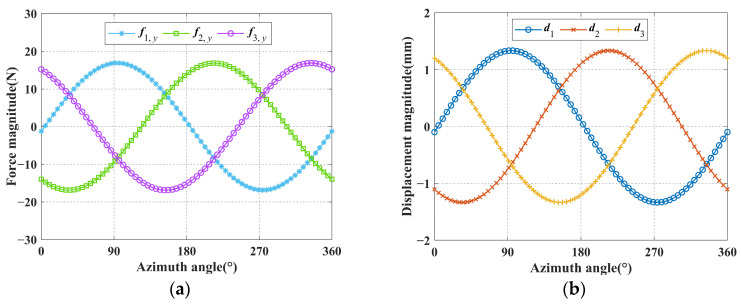
Curves of input forces and input displacements: (**a**) Curves of input forces; (**b**) Curves of input displacements.

For the convenience of defining spatial pointing, the coordinate frame *O*′*xyz* is established at point *O*′ and aligned with the global coordinate frame, as shown in Figure 5. Figure 5 shows that the spatial pointing of the mechanism can be represented by the normal vector *l_EO_* of mobile platform plane, where point *O* is the mobile platform center, and point *E* is the intersection of the *y*-axis in the coordinate frame *O*′*xyz* with the normal line of the mobile platform plane. The angle between the *z*-axis in the coordinate frame *O*′*xyz* and the projection of the normal vector *l_EO_* in the plane *O*′*xz* is defined as the azimuth angle *α*, and the angle between the *y*-axis in the coordinate frame *O*′*xyz* and the normal vector *l_EO_* is defined as the pitch angle *Ψ*. Then, the spatial pointing of the mechanism can be expressed in the terms of azimuth angle and pitch angle (*α*, *Ψ*). In this given spatial pointing trajectory, the azimuth angle *α* is discretized into 72 sampling points in the range of [0, 360°] and the pitch angle *Ψ* is 0.05 rad. Since the angular displacements (*θ_x_* and *θ_z_*) of the mobile platform in the Equations (12) and (26) are expressed in the form of RPY angles, the azimuth angle *α* and pitch angle *Ψ* in the spatial pointing trajectory need to be converted to the form of RPY angles in advance, and the conversion formula is given in Equation (75). Substituting the conversion results into Equations (12) and (26), the input forces and input displacements can be inversely calculated, respectively. Since both Equations (12) and (26) are mathematical models of underdetermined equations, infinite groups of input forces and input displacements solutions can be obtained theoretically. For ease of calculation, the minimum norm solution is used in this example, and the 72 sets of output angular displacements (*θ_x_* and *θ_z_*) were successively substituted into Equations (12) and (26) to obtain the corresponding input forces and input displacements. The curves of input forces and displacements are demonstrated in Figure 6a,b, respectively.

(75) $$rotz(\theta_{z})\cdot rotx(\theta_{x})\cdot \eta =roty(\alpha )\cdot rotx(\psi )\cdot \eta \;,\theta_{x},\theta_{z}\in \left[-\frac{\pi }{2},\frac{\pi }{2}\right]$$

**Figure 7:**
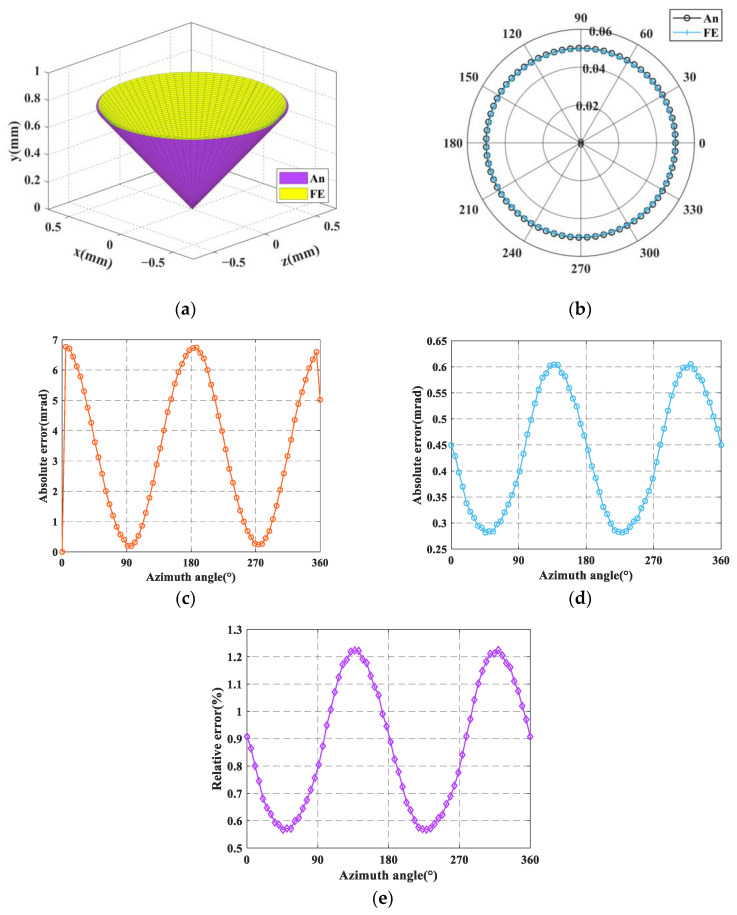
Comparison of analytical and FE results when the input type is the input force: (**a**) Comparison of spatial pointing between analytical results and FE-results; (**b**) Comparison of azimuth and pitch between analytical results and FE-results; (**c**) The absolute error of azimuth angle; (**d**) The absolute error of pitch angle; (**e**) The relative error of pitch angle.

**Figure 8:**
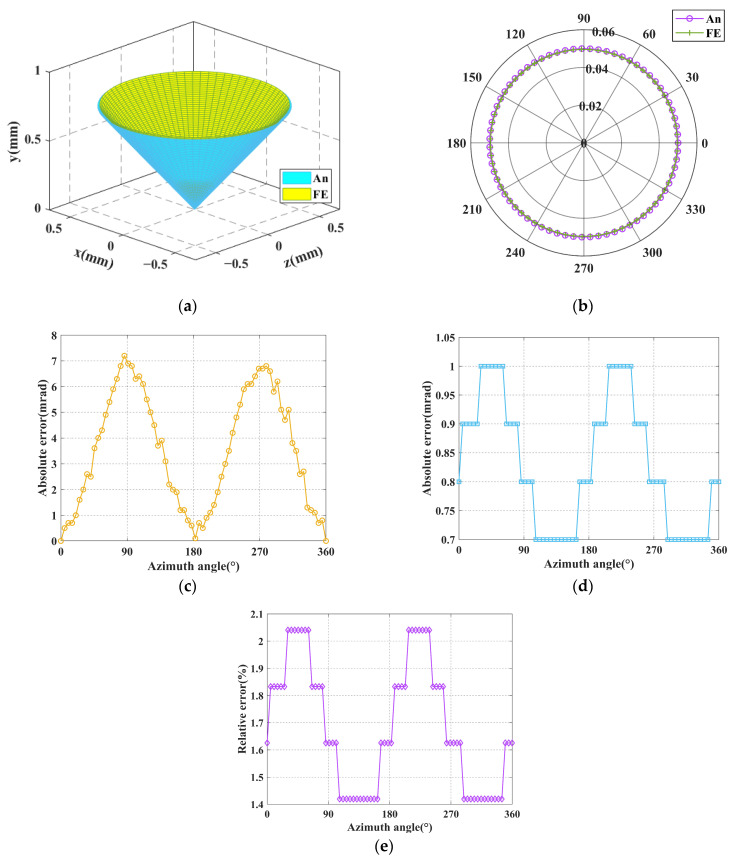
Comparison of analytical and FE results when the input type is the input displacement: (**a**) Comparison of spatial pointing between analytical results and FE-results; (**b**) Comparison of azimuth and pitch between analytical results and FE-results; (**c**) The absolute error of azimuth angle; (**d**) The absolute error of pitch angle; (**e**) The relative error of pitch angle.

The resulting input forces and displacements are successively substituted into the FE-model for simulation, and the simulated output angular displacements (*θ_x_* and *θ_z_*) of the mobile platform can be obtained. In Figure 7a and Figure 8a, the inner and outer cones represent the spatial pointing of the analytical results and FE-results (for ease of observation, the pitch angle in the picture is enlarged by 14 times), respectively. The comparison of the spatial pointing azimuth angle and the pitch angle between the analytical results and FE-results is demonstrated in Figure 7b and Figure 8b, in which the polar radius and angle represent the pitch angle and azimuth angle of the spatial pointing, respectively. The absolute error of the azimuth angle between the analytical results and FE-results are shown in Figure 7c and Figure 8c. The absolute error of the pitch angle between the analytical results and FE-results are presented in Figure 7d and Figure 8d. The relative error of the pitch angle between the analytical results and FE-results are presented in Figure 7e and Figure 8e.

It can be seen from Figure 7 that when the input type is the input displacement, the analytical results of spatial pointing are very close to the FE-results. The maximum absolute errors of azimuth angle and pitch angle are less than 7 mrad and 0.65 mrad, respectively, and the maximum relative error of pitch angle is less than 1.3%. It can be seen from Figure 8 that when the input type is the input force, the analytical results of spatial pointing are very close to the FE-results. The maximum absolute errors of azimuth angle and pitch angle are less than 8 mrad and 1.05 mrad, respectively, and the maximum relative error of pitch angle is less than 2.1%. The high consistency between the analytical results and the FE-results demonstrates the correctness of the kinetostatic model. In addition, when using the compliance matrix method to establish the kinetostatic model of the mechanism, the rigid rods are regarded as absolute rigid bodies, that is, they will not generate any elastic deformation. However, during the process of finite element simulation, the rigid rods will produce corresponding elastic deformation due to their own material properties. For this reason, there will be a certain degree of relative error between the theoretical calculation results and the simulation calculation results.

### 5.2. Calculation of Workspace

In this section, the Monte Carlo method was employed to obtain the workspace of the mechanisms. The calculation process is as follows: 1. The input force and input displacement are discretized into several sampling points within the value range, respectively. The maximum input force *F_max_* and maximum motion range *d_max_* selected in this paper are 30 N and 15 mm, respectively. 2. All the sampling points were substituted into the kinetostatic model and the flexible hinge displacement model, respectively, and the flexible hinge displacement and the output displacement of mechanism corresponding to different input types are obtained. 3. The maximum flexible hinge displacement is taken as the constraints, as shown in Table 3. When the flexible hinge displacement in any direction exceeds the constraints, the corresponding output displacement data is eliminated. When the flexible hinge displacement in any direction does not exceed the constraints, the corresponding output displacement data is retained. The intersection of the displacement data retained under different input types is taken to obtain the workspace of the mechanism.

According to the direction of the output displacement of the *n*-4R CPPM, the workspace can be divided into two parts. The pointing workspace consists of output displacements in the directions *θ_x_*, *θ_y_*, and *θ_z_*, and the position workspace consists of output displacements in the directions *δ_x_*, *δ_y_* and *δ_z_*. Since the *θ_y_*-direction is the parasitic displacement direction, only the displacement in the functional directions (*θ_x_* and *θ_z_*) needs to be considered. *θ_x_* and *θ_z_* can be converted into azimuth angle *α* and pitch angle *ψ* to represent the pointing workspace. The reachable pointing workspace consists of all the pitch angles that can be reached by the mechanism in any azimuth (the maximum pitch angle *ψ_max_* in the reachable pointing workspace is simply referred to as the maximum achievable pitch angle *ψ_a_*). For a class of *n*-4R CPPMs, in order to avoid structural complexity during the design and selection process, the value of *n* should not be too large. Therefore, the 3-4R and 4-4R CPPMs are selected as examples to calculate the workspaces.

**Figure 9:**
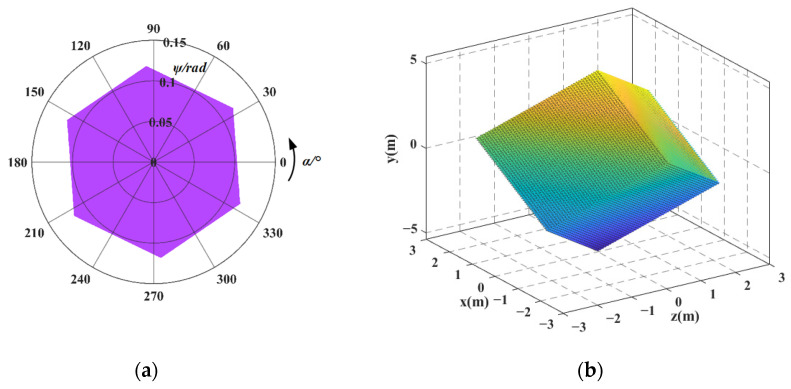
The workspace of 3-4R CPPM corresponding to the redundant actuated case: (**a**) pointing workspace; (**b**) position workspace.

**Figure 10:**
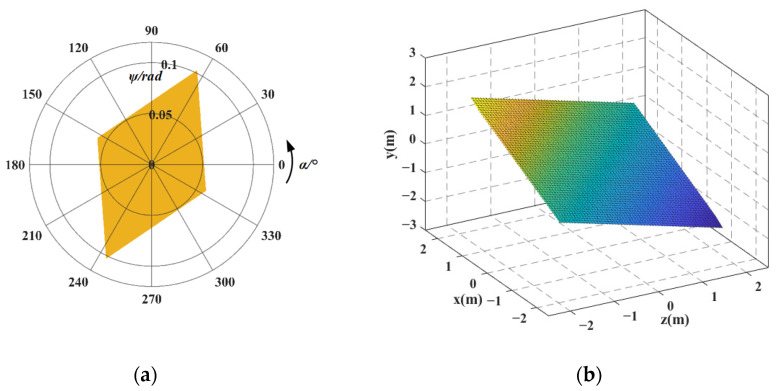
The workspace of 3-4R CPPM corresponding to the non-redundant actuated case: (**a**) pointing workspace; (**b**) position workspace.

**Figure 11:**
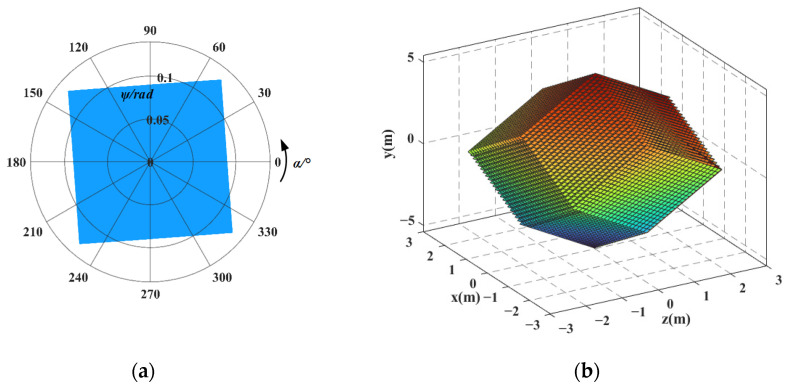
The workspace of 4-4R CPPM corresponding to the redundant actuated case: (**a**) pointing workspace; (**b**) position workspace.

**Figure 12:**
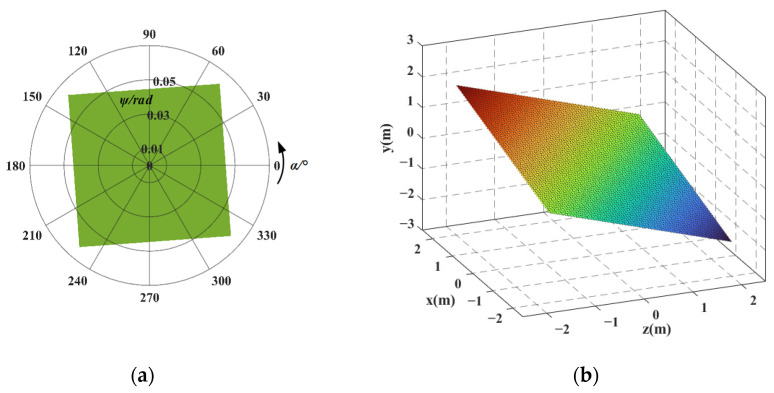
The workspace of 4-4R CPPM corresponding to the non-redundant actuated case: (**a**) pointing workspace; (**b**) position workspace.

First, taking the redundant actuated 3-4R CPPM as an example, each input force ***F****_i_* = [0, 0, 0, 0, *f*_*i*,*y*_, 0]^T^ (*i* = 1, ..., 3) and input displacement $U_{F_{i}\_total}$ = [0, 0, 0, 0, *d_i_*, 0]^T^ (*i* = 1, ..., 3) along the *y*-axis direction are discretized into 200 sampling points within the value range. Then, each set of input forces and displacements are successively substituted into Equations (11), (24), (59) and (60), respectively, and the output displacement of mechanism and flexible hinge displacement are obtained. Finally, taking the maximum flexible hinge displacement as the constraint condition, the output displacement data that does not meet the constraint condition is eliminated. The workspace is obtained by taking the intersection of the output displacements of different input types in the retained data, as shown in Figure 9. For ease of comparison, the workspace of the 3-4R CPPM corresponding to the non-redundant actuated case is shown in Figure 10. In Figure 9a and Figure 10a, the polar diameter represents the pitch angle, and the polar angle indicates the azimuth angle. The reachable pointing workspace can be regarded as the inscribed circle of pointing workspace. Similarly, each set of input force ***F****_i_* = [0, 0, 0, 0, *f*_*i*,*y*_, 0]^T^ (*i* = 1, ..., 4) and input displacement $U_{F_{i}\_total}$ = [0, 0, 0, 0, *d_i_*, 0]^T^ obtained discretely can be successively substituted into Equations (31), (39), (73), and (74), respectively, and the workspaces of the redundant actuated 4-4R CPPM and the non-redundant actuated case are obtained, as shown in Figure 11 and Figure 12. The comparison results of 3-4R and 4-4R CPPMs workspaces are shown in Table 4.

By comparing Figure 9 and Figure 10 and the results in Table 4, it can be seen that the workspace of 3-4R CPPM has the following changes by means of the redundant actuation: (1) The pointing-workspace changes from a quadrilateral to a hexagonal. In the case of non-redundant actuation, due to the geometric characteristics, the maximum pitch angle is not distributed at all vertices of the quadrilateral, but only at the upper and lower two acute vertices. In the case of redundant actuation, the maximum pitch angle is distributed at the six vertices of the hexagon. It should be noted that the maximum pitch angles are achievable only at a few special azimuths. (2) The position-workspace changes from an oblique quadrilateral in space to a hexahedron, and the achievable motion range of the mechanism in the *x* and *z* directions becomes the same, making the motion of the mechanism more symmetrical. (3) The maximum pitch angle *ψ_max_* is increased by 0.015 rad, the maximum achievable pitch angle *ψ_a_* is increased by 0.051rad, and the motion ranges in the *x*, *y*, and *z* directions (*x_d_*, *y_d_* and *z_d_*) are increased by 0.86 mm, 0.54 mm, and 0.34 mm, respectively. Among them, the motion range in the *y*-direction and the maximum achievable pitch angle increased the most, both 100%. (4) The workspace in the non-redundant actuated case is a subset of the workspace in the redundant actuated case.

By comparing Figure 11 and Figure 12 and the results in Table 4, it can be seen that the workspace of 4-4R CPPM has the following changes by means of the redundant actuation: (1) The position workspace changes from an oblique rectangle in space to a spatial dodecahedron, and the achievable motion range of the mechanism in the *x* and *z* directions becomes the same, also making the motion of the mechanism more symmetrical. (2) The maximum pitch angle *ψ_max_* is increased by 0.063 rad, the maximum achievable pitch angle *ψ_a_* is increased by 0.044 rad, and the motion ranges in the *x*, *y*, and *z* directions are increased by 0.54 mm, 0.5 mm, and 0.58 mm, respectively. Among them, the motion range in the *y*-direction, the maximum achievable pitch angle, and maximum pitch angle increased the most, both 100%. (3) The workspace in the non-redundant actuated case is a subset of the workspace in the redundant actuated case.

### 5.3. Analysis of Workspace

In Section 5.2, the workspace of 3-4R and 4-4R CPPMs are obtained. It can be seen that, compared with the non-redundant actuation case, the size and shape of the mechanism’s workspace changed. In this section, the changes in the workspace will be further analyzed.

(1) In the non-redundant actuated case, the actuation forces are only exerted on two adjacent branches of the mechanism, and the forces distribution within the mechanism is non-uniform. Therefore, the 3-4R and 4-4R CPPMs have different motion ranges in the *x* and *z* directions within the position workspace. Moreover, the maximum pitch angles of 3-4R CPPM is only at the two vertices of the quadrilateral-shaped pointing workspace, rather than at all four vertices. The 4-4R CPPM features orthogonal symmetry. As a result, the pointing workspace is rectangular, and the maximum pitch angles are evenly distributed at the four vertices. In the redundant actuated case, the actuation forces are exerted on each branch of the mechanism, resulting in a more even force distribution. Consequently, the motion ranges of the 3-4R and 4-4R CPPMs in the *x* and *z* directions become the same. In addition, the shape of the 3-4R CPPM pointing workspace changes to a hexagon, and the maximum pitch angles are distributed at the six vertices of pointing workspace. However, the shape of the 4-4R CPPM pointing workspace remains unchanged. The result indicates that, with the redundant actuation, the actuation forces are evenly exerted on each branch of the mechanism, and the workspace can be made more symmetrical. Moreover, since the shape of the 4-4R CPPM pointing workspace remains unchanged, it indicates that the orthogonal symmetry has an impact on the shape of the mechanism’s workspace.

(2) In the non-redundant actuated case, the relationship between output and input is that each output displacement corresponds to a unique input force/displacement. If the flexible hinge displacement does not meet the constraint conditions, the mechanism cannot reach the corresponding output position. In the redundant actuated case, the relationship between output and input is that each output displacement corresponds to an infinite set of input forces/displacements, and an infinite set of inputs corresponds to an infinite set of flexible hinge displacements. It is possible to find a set of inputs among the infinite sets of inputs so that the flexible hinge displacement meets the constraint conditions. Some output positions that cannot be reached in the non-redundant actuated case are reached by means of the redundant actuation, and thus, the workspace of the mechanism is increased. For the above-mentioned reasons, the workspace in the non-redundant actuated case also becomes a subset of the workspace in the redundant actuated case. Since the orthogonal symmetry of the 4-4R CPPM, the maximum pitch angle is increased by 100%. This indicates that the orthogonal symmetry also affects the size of the mechanism’s workspace.

(3) In the non-redundant actuated case, each output displacement in the *x* and *z* directions corresponds to the unique output displacement in the *y*-direction. Therefore, the position workspaces of the 3-4R and 4-4R CPPMs are planar shapes. In the redundant actuated case, each output displacement in the *x* and *z* directions corresponds to a non-unique output displacement in the *y*-direction. Thus, the position workspaces of the 3-4R and 4-4R CPPMs have changed into 3-D shapes.

It should be noted that the structural parameters of the mechanism also have an important impact on its performance, and optimizing these parameters has the potential to enhance the workspace performance of the mechanism. In this paper, the method of improving the mechanism’s workspace performance from the perspective of introducing redundant actuation, and the implementation process of optimization does not involve changes in structural parameters. In practice, the proposed optimization method can be carried out after the optimization of the structural parameters. Therefore, the effect based on the redundant actuation can be superimposed with that of the structural parameter optimization.

## 6. Conclusions

This paper introduces a class of redundant actuated *n*-4R CPPM, and relevant conclusions are summarized as follows.

(1) The kinetostatic model of the redundant actuated *n*-4R CPPM is established to reveal the mapping relationship between the mobile platform’s output displacements and the input forces/displacements of the actuators. The kinetostatic model of the redundant actuated 3-4R CPPM is verified through the comparison between the analytical calculation and FE-simulation using a given space pointing trajectory. The results demonstrate that when the input types are input force and displacement, the relative errors between the analytical results and the FE-results are no more than 1.3% and 2.1%, respectively, and the high consistency validates the kinetostatic model.

(2) The flexible hinge displacement model of the redundant actuated *n*-4R CPPM is established to reveal the deformation of the flexible hinge in six directions during the mechanism’s motion, thereby facilitating a more accurate calculation of the workspace.

(3) Taking the maximum displacement of the flexible hinge and the maximum range of the actuator as constraints, combining the kinetostatic and flexible hinge displacement model of the mechanism, the workspace of the 3-4R and 4-4R CPPMs in redundant actuation and non-redundant actuation cases is obtained, respectively. The results show that compared with the non-redundant actuation case, the workspace can be increased and becomes more symmetrical by means of the redundant actuation, and the results are universal. For both the 3-4R and 4-4R CPPMs, the maximum achievable pitch angle and the motion range in the *y*-direction increase by 100%, and the motion ranges in the *x* and *z* directions become the same. Due to the orthogonal symmetry of 4-4R CPPM, the maximum pitch angle is also increased by 100%. Furthermore, the workspace in the non-redundant actuated case is a subset of the workspace in the redundant actuated case, and the shape of position workspace changes from planar to 3-D.

## Figures and Tables

**Table 1:** The parameters of coordinate transformations.

	*x*	*y*	*z*	*θ_x_*	*θ_y_*	*θ_z_*
$Ad_{O_{11}}^{O}$	$-r\cos\frac{\varphi }{2}$	$r\sin\frac{\varphi }{2}-L$	*R*	0	0	$\pi -\frac{\varphi }{2}$
$Ad_{O_{12}}^{O}$	$r\sin\frac{\varphi }{2}-l$	$l\tan\frac{\varphi }{2}+r\cos\frac{\varphi }{2}-L$	0	$-\frac{\pi }{2}$	$\frac{\left(\varphi -\pi \right)}{2}$	0

**Table 2:** Parameters of the 3-4R CPPM.

Hinge Parameters	Values	Structural Parameters	Values
*E*/pa	2.06 × 10^11^	*l*/mm	66.6
*μ*	0.3	*φ/*°	60°
*t*_0_/mm	0.5	*R*/mm	66
*r*/mm	3.75	*dx*/mm	41.006
*w*/mm	5	*dy*/mm	88.308
		*dz*/mm	59
		*L*/mm	100

**Table 3:** Maximum displacement of flexible hinge.

Maximum angular displacement	$\theta_{x\max}$ (rad)	$\theta_{y\max}$ (rad)	$\theta_{z\max}$ (rad)
	0.0124	0.00562	0.0253
Maximum line displacement	$\delta_{x\max}$ (mm)	$\delta_{y\max}$ (mm)	$\delta_{z\max}$ (mm)
	7.962 × 10^−3^	9.671 × 10^−2^	3.136 × 10^−2^

**Table 4:** Comparison results of 3-4R and 4-4R CPPMs workspaces.

Name of the CPPM	3-4R		4-4R	
Actuation Case	Redundant	Non-Redundant	Redundant	Non-Redundant
*ψ_max_*/rad	0.118	0.103	0.126	0.063
*ψ_a_*/rad	0.102	0.051	0.088	0.044
*x_d_*/mm	−2.7~2.7	−2.27~2.27	−2.34~2.34	−2.07~2.07
*y_d_*/mm	−0.54~0.54	−0.27~0.27	−0.5~0.5	−0.25~0.25
*z_d_*/mm	−2.7~2.7	−2.53~2.53	−2.34~2.34	−2.05~2.05

## Data Availability

All data that support the findings of this study are included within the article.
